# Associations Between Parental Alcohol Use and Parenting Practices: A Systematic Review

**DOI:** 10.3390/bs16020236

**Published:** 2026-02-07

**Authors:** Barbara Oliveira Carvalho, Tonje Holte Stea, Lindsey Coombes, Siri Håvås Haugland

**Affiliations:** 1 Department of Psychosocial Health, University of Agder, 4898 Grimstad, Norway; siri.h.haugland@uia.no; 2 Department of Health and Nursing Science, University of Agder, 4604 Kristiansand, Norway; tonje.h.stea@uia.no; 3 Department of Psychology, Social Work and Public Health, Oxford Brookes University, Oxford OX3 0BP, UK

**Keywords:** parenting, parental alcohol use, alcohol use disorders, non-dependent drinking, parenting practices, general parenting, alcohol-specific parenting, parent–child relationship

## Abstract

Parental practices strongly influence offspring development, and parental alcohol use may affect parenting behavior. However, most studies have focused on child-related outcomes. This review instead examined associations between parental drinking and parenting practices. Following PRISMA guidelines, a systematic search in MEDLINE, PsycINFO, Embase, and Scopus identified 9053 articles. Of these, 222 full texts were screened by two reviewers, and 77 were included for critical appraisal. After quality assessment, 68 studies published between 1991 and 2026 were reviewed. Studies were included if they (i) measured parental alcohol use as a predictor and parenting practices as an outcome, (ii) involved offspring under 20 years, (iii) had a quantitative design, (iv) were peer-reviewed and published in English, and (v) excluded pregnancy measures and alcohol/parenting treatment interventions. Parenting factors (e.g., parent–child relationship, monitoring, communication, discipline, conflict, abuse) were categorized and grouped into general and alcohol-specific practices. Parental drinking was categorized into alcohol use disorders (AUD) and non-dependent alcohol use. Sixty-four studies reported at least one significant association between parental drinking and poorer parenting practices. Mixed results were observed for non-dependent drinking and alcohol-specific parenting. Some associations varied with parental gender. Overall, different drinking patterns appear to be linked to impaired parenting quality. Despite being the most common pattern of alcohol use, non-dependent drinking remains understudied in parenting research. The present review highlights several critical gaps in the literature, particularly regarding the relationship between non-dependent drinking, maternal AUD, and parenting practices. Moreover, contextual factors, such as socioeconomic status and gender differences, are insufficiently explored, limiting the understanding of heterogeneity in risk and outcomes. Future research would benefit from employing robust longitudinal designs and expanding geographical representation, in order to capture variation across sociocultural contexts and enhance the generalizability of findings.

## 1. Introduction

The World Health Organization recently called for accelerated action to implement a global strategy to reduce the harmful use of alcohol as a public health priority and to improve the health and social outcomes of individuals, families, and communities. This call reinforces earlier concerns about alcohol’s harm to others (World Health Organization, 2022). As alcohol consumption often occurs in the family context, it has the potential to inflict collateral damage beyond the drinker (Haverfield & Theiss, 2014). Indeed, in high-income countries, between 5% and 30% of children are estimated to live in the same household as parents with problematic alcohol use (McGovern et al., 2020). Multiple negative outcomes are related to alcohol’s harm to others, such as adverse experiences, violence, injury, mental health problems, the increased risk of substance use, and disease (Laslett et al., 2019; World Health Organization, 2022). Studies have found positive associations between parents’ drinking behavior and parenting practices, on the one hand, and the development of similar alcohol patterns in their offspring, on the other (Latendresse et al., 2008; Yap et al., 2017). Moreover, children who have two parents with alcohol use disorder (AUD) are at a higher risk of abuse, violence, neglect, and externalizing behaviors than those with one alcoholic parent (Anda et al., 2002; Dube et al., 2001). Additionally, parental AUD may represent a burden to the family in periods with less drinking or alcohol withdrawal due to the consequences associated with AUD (e.g., health loss and behavioral and emotional problems), which demands attention throughout the drinking cycle (Griswold et al., 2018; National Institute on Alcohol Abuse and Alcoholism, 2021).

Parents have a crucial influence on the development of their offspring, and their parenting practices are highly relevant to the well-being of their children (Latendresse et al., 2008; Yap et al., 2017). Parenting has previously been categorized into four broad styles: authoritative, authoritarian, permissive, and neglectful (Maccoby & Martin, 1983). However, the rising sensitivity to cultural and contextual variations has led to a shift toward research examining parenting in terms of dimensions instead of these broad styles (Smetana, 2017).

While classical categorizations are still used, researchers also report on parenting dimensions, such as warmth, emotional support, control, and monitoring (Maccoby & Martin, 1983). However, the lack of a generally acknowledged standard method of conceptualizing and defining parenting practices or styles may limit the comparability of the results between studies. Further, the definitions of parenting factors in the literature lack consistency and frequently overlap conceptually, lowering our understanding of which specific parenting practices can be protective and beneficial for the child (Ryan et al., 2010).

Beyond general parenting, research has focused on alcohol-specific parenting, which covers parenting behaviors that discourage their children from consuming alcohol, or indeed encourage them to do so (Handley & Chassin, 2013; Mares et al., 2013). The literature has shown that strict alcohol-specific rule-setting seems to prevent adolescent alcohol use or at least reduce its frequency and intensity (Mares et al., 2012; Van der Vorst et al., 2007). By contrast, the results on the potential protective impact of parent–child alcohol-specific communication on alcohol drinking are mixed (Ennett et al., 2001; Mares et al., 2011). More frequent alcohol-specific communication has been shown to predict reduced adolescent drinking (Mares et al., 2011) as well as increase adolescent alcohol use (Ennett et al., 2001). Nevertheless, the distinction between frequency and quality in alcohol-specific communication should be made, as the quality of parental alcohol-related discussions can deter adolescent alcohol use (Mares et al., 2013).

Previous research has further indicated that parental alcohol use may negatively impact a range of parenting practices such as monitoring, control, positive parenting, support, structure, parent–child interaction, relationship quality, warmth, parental discipline, and alcohol-specific parenting behaviors (Chassin et al., 1993; Lang et al., 1999; Latendresse et al., 2008; McGovern et al., 2020; Van der Vorst et al., 2006). Chassin et al. (1993) found that maternal alcohol use disorder was associated with lower levels of both maternal and paternal monitoring of children. Alcohol-related problems can disrupt the quality of parent–child relationships, contributing to harsher parenting practices and diminished levels of supervision (McGovern et al., 2020). Similarly, Latendresse et al. (2008) further observed that parental alcohol use was negatively associated with shared activities and monitoring, while positively linked to increased relational tension and stricter disciplinary practices.

Additionally, Van der Vorst et al. (2006) noted that parental alcohol consumption was also associated with fewer and less strict rules regarding adolescent drinking, with parents who consume higher amounts of alcohol tending to be more permissive about their children’s alcohol use. Experimental research by Lang et al. (1999) indicated that intoxicated parents displayed less attention and engaged in inconsistent parenting, issuing more commands while simultaneously exhibiting more indulgent behaviors.

Although clinical diagnosed alcohol consumers have received widespread attention from previous research, they do not account for all or even most of alcohol’s collateral damage (Rossow et al., 2016). A significantly larger number of children are likely to be exposed to parental alcohol use that falls below clinical criteria for dependence (McGovern et al., 2020). However, less is known about how non-dependent parental alcohol use patterns, such as drinking at lower levels and heavy episodic drinking, relate to parenting and affect the children exposed to them (Rossow et al., 2016). Moreover, non-dependent consumption patterns are much harder to identify, which can jeopardize the potential of early intervention and prevention (McGovern et al., 2020).

This systematic review studies the relationship between parental alcohol use (AUD and non-dependent drinking) and parenting practices (general and alcohol-specific). The specific objectives are to (1) identify and provide an overview of studies that investigate the associations between parental alcohol use and parenting practices, (2) summarize the evidence obtained from the identified studies and synthesize the relevant findings, and (3) identify the limitations and gaps in the literature as a basis for further studies. A review of the available research can contribute important knowledge to understand why adverse consequences follow parental alcohol use.

## 2. Materials and Methods

The systematic review followed Preferred Reporting Items for Systematic Reviews and Meta-Analyses guidelines (PRISMA) and the protocol was registered on PROSPERO (registration number: CRD42020153650). PRISMA checklists in the Supplementary Materials (Tables S1 and S2).

### 2.1. Search Strategy

The most recent systematic literature search was conducted on 6 January 2026. The initial search was carried out on 12 April 2019 across four online databases (MEDLINE, PsycINFO, Embase via Ovid, and Scopus) and subsequently updated on 6 December 2021 using the same strategy for all searches. The database search strategy combined terms across parental alcohol drinking as well as parenting practices and factors. Index terms such as MeSH and Thesaurus and text words were applied. No restrictions on publication date or language were imposed. Appendix A presents the search strategy used for Ovid databases. Further searches of studies were performed by checking the reference lists of the included papers and forward searching using Google Scholar and the Science Citation Index. We contacted experts to identify additional studies that we might have missed.

The main search was conducted by the leading reviewer (BOC), assisted by an experienced academic librarian, and all the studies were imported into EndNote and Covidence software for systematic review management. After removing duplicates, all the titles and abstracts were screened, and eligible studies that met the inclusion and exclusion criteria were included. A full-text assessment was performed for each eligible study identified as potentially relevant for the review. To prevent bias, two reviewers performed the title and abstract screening and full-text assessments independently (BOC, SHH and THS). Discrepancies were discussed, and any disagreements on selection, quality assessment, and data collection were resolved by other reviewers.

### 2.2. Selection Criteria

Studies were included in the review if they used a quantitative study design, had a population of parents with offspring aged 0 to 19 when exposed, measured parental alcohol consumption (AUD and non-dependent drinking patterns) as a predictor variable, and parenting practices (general and alcohol-specific) as an outcome. Appendix B presents the definitions of parenting variables. Data on parental alcohol consumption and parenting practices could be obtained from either parents, children, or official records. Parental data could include information on both parents or either parent, including biological and non-biological parents. Searches were not restricted to English-language papers, though studies were only eligible if they were published in a peer-reviewed journal with a full-text in English. Neither geographic limitations nor date restrictions were applied.

We excluded all studies that did not meet any of the above criteria. Furthermore, studies were excluded if parental alcohol use was measured during pregnancy, if the independent and dependent variables were not related to the same individual/parent, and if participants were receiving interventions that might influence the results, such as treatment for alcoholism or addiction and participation in parent training programs.

Only studies reporting the direct associations or effects between parental alcohol use and parenting factors (general and alcohol-specific) were included in the final review. Studies combining distinct constructs, as either predictor or outcome variables, were also excluded (e.g., studies in which parental alcohol use was combined into a composite measure with other substance use).

### 2.3. Quality Assessment

The methodological quality of the included studies was assessed to establish their internal validity and risk of bias. This assessment was conducted independently by two reviewers (BOC and SHH) using guidance from the Critical Appraisal Skills Programme (CASP). A template was created in the Covidence software based on CASP checklists suitable for the study design. Considering the importance of both the quality and the reliability of the findings of the included studies on the systematic review’s internal validity (Carroll et al., 2012), the authors excluded nine studies following the quality assessment due to high risk of bias (see Tables S3 and S4 in Supplementary Material for more information and the main reasons). Studies with an overall low or moderate risk of bias were included.

### 2.4. Data Synthesis

The expected and noticeable heterogeneity of the studies (different samples, measures, and designs) prevented us from conducting a meta-analysis; therefore, a narrative synthesis was carried out by the main reviewer (BOC) and revisited by SHH. We explored the relationships and findings both within and between the included studies. Tables were used to present the findings of the included studies and identify patterns across the studies to facilitate the narrative synthesis.

The following categories of information were extracted for the data synthesis: publication details (author(s), reference, publication date, and country), study characteristics (study design, methodology, sample, exposure and outcome variables, measures, and confounders), participant characteristics, and results (findings, parental gender, offspring age, and statistical method).

## 3. Results

**Figure 1:**
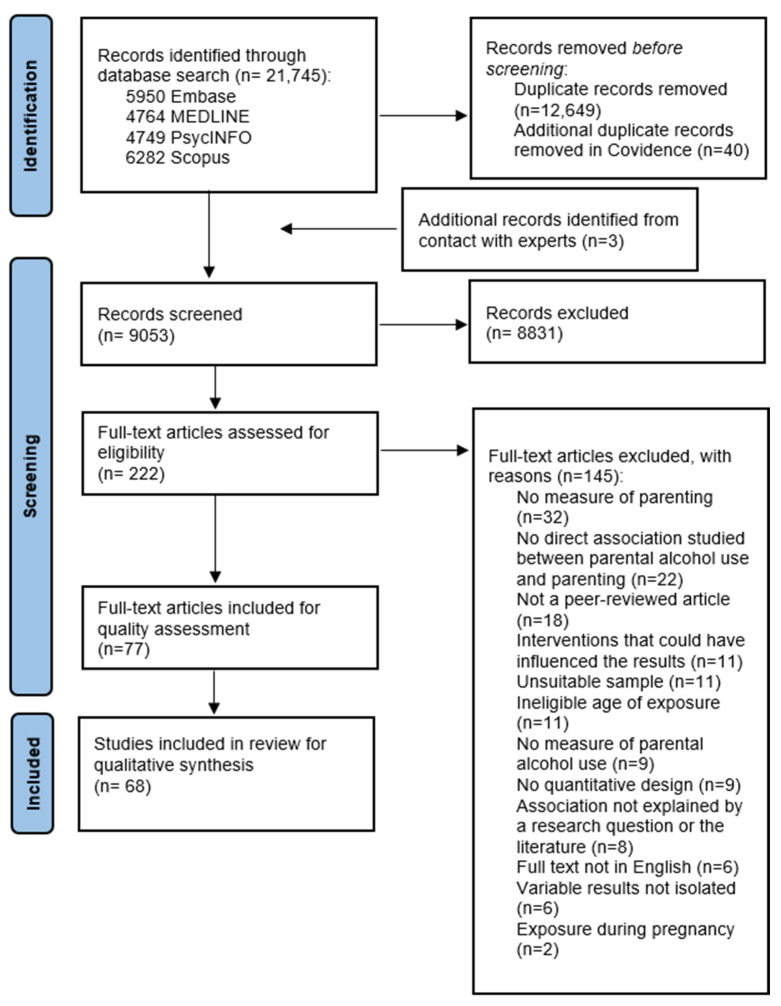
PRISMA flowchart of study selection summarizes the data collection process in the PRISMA flowchart (we also followed PRISMA guidance on reporting).

The PRISMA flowchart (Figure 1) illustrates the systematic literature search process. After removing duplicates, 9053 titles and abstracts were screened, of which 222 papers were assessed based on the full texts. Of these, 77 studies met the inclusion criteria. As noted above, critical appraisal led to the exclusion of nine studies, leaving 68 studies included in the narrative synthesis (List S1, and Table S4 in Supplementary Material).

Table 1A,B describe the characteristics (e.g., author(s), date, study design, sample, and exposure and outcome variables) and main findings of these 68 studies divided by alcohol exposure: alcohol use disorder (AUD) and non-dependent alcohol use. The sample size ranged from 161 mothers to 10,210 families. A total of 36 of the included studies involved children (0–12 years), whereas 13 studies studied adolescents (13–19 years), and 12 studies included both age groups. One study did not specify the age of the offspring and reported only the fathers’ ages, despite referring to the offspring as children (Laslett et al., 2022). Furthermore, the results of six studies were reported retrospectively by the adult children of parents with alcohol problems. The studies were conducted on general population samples, without specifically targeting groups characterized by behavioral problems, medical conditions, or other special attributes. Clinical or special needs populations were excluded, with the exception of the study by Lang et al. (1999), which compared parents of children with externalizing behavior problems to a control group. The included studies were published between 1991 and 2026 and conducted in fourteen countries. The majority was conducted in the United States (*n* = 53), with nine studies in Europe (Netherlands *n* = 3, United Kingdom *n* = 2, Belgium *n* = 1, Czech Republic *n* = 1, Ireland *n* = 1, Norway *n* = 1), two each in Australia and China, and one in South Africa. One study gathered participants from five Asia-Pacific region countries: Cambodia, China, Indonesia, Papua New Guinea, and Sri Lanka (Laslett et al., 2022).

The comparison of results from included studies revealed that some were based on samples obtained from the same record or project. A larger group of papers used data from New York State birth records for Erie County (Edwards et al., 2004, 2009; Eiden et al., 1999, 2009a, 2002, 2004a, 2007; Eiden & Leonard, 2000; Eiden et al., 2004b, 2009b; Finger et al., 2010; Kachadourian et al., 2009). An earlier project (Jacob et al., 1989) on drinking and family interaction served as a reference for other studies (Jacob et al., 1991, 2001; Moser & Jacob, 1997). Data collected from the Dutch Family and Health longitudinal survey, which examines the socialization processes underlying adolescents’ health behaviors, were also applied (Mares et al., 2011; Van der Vorst et al., 2006; Van der Zwaluw et al., 2008). Participants were further sampled from a general population study of parents in middle-sized cities in California (Freisthler et al., 2014, 2015; Freisthler & Wolf, 2016; Lloyd & Kepple, 2017).

More studies (*n* = 40) focused on dependent drinking patterns (i.e., meeting the diagnostic criteria for AUD), while the remaining 28 measured non-dependent drinking patterns (e.g., frequency, binge drinking, and heavy episodic drinking).

In 57 of the 68 studies, the outcomes were related to general parenting practices. Nine studies examined alcohol-specific parenting practices only, and two others included both general and alcohol-specific parenting outcomes. Of the eleven studies addressing alcohol-specific parenting, three focused on AUD (including the two that examined both parenting categories), while the remaining eight investigated non-dependent alcohol drinking patterns.

Overall, most studies (64 of the 68) reported at least one direct and significant association between parental alcohol use and impaired parenting practices. Of the four studies that found no statistically significant direct association, one investigated parental supply of alcohol (Ward & Snow, 2011) and another was an experimental study of alcohol intake observed during family interactions (Jacob et al., 1991). The remaining two studies examined general parenting practices such as harsh parenting and supervisory neglect. Although they found no direct effect when combined with drinking, the effect was significant when such variables as marital aggression and depressive symptoms or decreased social support were introduced (Bijttebier & Goethals, 2006; Lloyd & Kepple, 2017).

## 4. Discussion

To the best of our knowledge, this is the first systematic review to examine the associations between parental alcohol use and parenting practices. Overall, we found consistent evidence across 64 of the 68 included studies, supporting the finding that increased parental alcohol use (AUD and non-dependent drinking patterns) was associated with impaired or reduced quality parenting practices (general and alcohol-specific parenting behaviors). Some of these associations varied with parental gender. More of the studies investigated the extent to which parental AUD was associated with general parenting practices than non-dependent drinking patterns and general parenting practices. However, regarding alcohol-specific parenting, most of the studies investigated non-dependent consumption.

### 4.1. Parental AUD and Parenting Practices

#### 4.1.1. General Parenting

Six studies showed that parents with AUD have an increased risk of displaying abusive or harsh parenting behavior (Anda et al., 2002; Eiden et al., 2009b; Famularo et al., 1992; Finger et al., 2010; Jacques et al., 2020; Keller et al., 2021). It is well established that alcohol use may increase aggression, conflict, and domestic violence (Dube et al., 2001; Laslett et al., 2012; Schacht et al., 2009; Seay, 2026; Senchak et al., 1995; Tweed & Ryff, 1996), and Finger et al. (2010) also suggested that the pathway from paternal AUD to harsh parenting goes through marital aggression. Several mechanisms of why parental AUD may increase the risk of violence toward offspring have been suggested (Miller et al., 1997). First, intoxication may affect and restrict the perception and capability of dealing with information. Social cues and communication with family members may be disturbed if only parts of the social interaction are caught by the drinker. Second, alcohol use can “allow” the drinker to commit offences and attribute the behavior to the alcohol use and, in this way, avoid being accountable for their actions. Third, alcohol is proposed to affect parts of the brain that regulate unacceptable behaviors, such as various forms of abuse.

A substantial number of studies (*n* = 24) showed that parents with AUD exhibited some form of reduced quality in the parent–child relationship compared with parents without AUD, such as less warmth, impaired attachment, and less positive interactions (Bijttebier & Goethals, 2006; Edwards et al., 2004; Eiden et al., 1999, 2009a, 2002, 2004a, 2007, 2004b; Eiden & Leonard, 2000; Jacob et al., 1991, 2001; Jacques et al., 2025, 2021; Kachadourian et al., 2009; Keller et al., 2024, 2021; Kelley et al., 2011; Moser & Jacob, 1997; Rangarajan, 2008; Rochat et al., 2019; Rutherford et al., 1998; Schacht et al., 2009; Senchak et al., 1995; Su et al., 2018; Tweed & Ryff, 1996).

During interactions with their children, parents with AUD also demonstrated less open communication (Keller et al., 2021; Ohannessian, 2012), lower positive engagement and sensitivity (Eiden et al., 1999, 2009a, 2002; Jacques et al., 2025, 2021), more negative attitudes, greater alienation, poorer communication, and greater mistrust (Finan et al., 2015; Keller et al., 2024; Kelley et al., 2011). Diminished parental monitoring was also related to parental AUD (Chassin et al., 1996, 1993; Elam et al., 2020; Taber-Thomas & Knutson, 2021; Van der Zwaluw et al., 2008), while the provision of less social support and inconsistent discipline was associated with maternal (Handley & Chassin, 2013; Jacques et al., 2025, 2020; Taber-Thomas & Knutson, 2021), but not paternal drinking (Handley & Chassin, 2013). In a study with both mothers and fathers, the relationship between AUD and supportive parenting was entirely indirect, mediated through parent depression (Keller et al., 2024).

AUD may hamper parenting practices due to periods of uncontrolled drinking, black-outs, alcohol craving, and embracing negative emotional states when not drinking (National Institute on Alcohol Abuse and Alcoholism, 2021). Intoxication and alcohol withdrawal symptoms can also cause anxiety, irritability, dysphoria, and a lack of motivation (McCrady & Flanagan, 2021; National Institute on Alcohol Abuse and Alcoholism, 2021). These problems typically hinder the performance of other responsibilities, decision-making abilities, and personal relationships, including parenting.

The findings of some of the included studies were less consistent on the associations between parental AUD and impaired general parenting (Finger et al., 2010; Jacob et al., 1991; Van der Zwaluw et al., 2008); some researchers only found associations for either mothers (Elam et al., 2020; Handley & Chassin, 2013; Van der Zwaluw et al., 2008) or fathers (Chassin et al., 1996; Rangarajan, 2008; Su et al., 2018). Additionally, the finding by Seay (2026) that problematic alcohol use was associated with increased parental monitoring was unexpected. Although compensatory parenting behaviors or parents’ own history of trauma could help explain this result, it is important to note that this specific finding was not confirmed in the second half of the dataset. The author acknowledges that the study may have had limited statistical power to detect the true effect size or that the relationship may be specific to this unique subset of families.

A small subset studies included in this review employed experimental or quasi-experimental designs to examine the short-term effects of parental alcohol consumption on observed parenting behaviors. Notably, three laboratory-based observational studies conducted by Jacob and colleagues (Jacob et al., 1991, 2001; Moser & Jacob, 1997) experimentally manipulated acute alcohol intoxication under controlled conditions and assessed parent–child and marital interactions during structured problem-solving tasks. These studies are exceptional within the broader literature and likely reflect an era of alcohol research in which controlled laboratory paradigms were used to isolate immediate behavioral effects of intoxication. Although these designs offered rare insights into mechanisms linking alcohol use to parenting behaviors, through direct observation and standardized coding of interaction patterns, they also raise important ethical concerns. In these studies, parents were administered alcohol in the presence of their children, and family conflicts were deliberately elicited using personally relevant topics, potentially exposing children to distressing interactions involving intoxicated caregivers. Despite implementation of safety procedures and strict time limits, such paradigms pose ethical challenges related to child welfare, psychological risk, and ecological validity. These concerns likely contribute to the scarcity and historical concentration of experimental studies in this area, as contemporary research ethics standards have increasingly constrained the feasibility of experimentally manipulating parental alcohol use in family contexts. Consequently, more recent research has relied predominantly on observational, longitudinal, and registry-based designs, which, while less able to establish short-term causal effects, better align with current ethical frameworks.

However, findings from one of these studies, (Jacob et al., 1991) provided only limited evidence that alcohol consumption alters parent–child interactions in families with a parent diagnosed with AUD, as the observed effects were not clearly distinguishable from those observed in control groups. The authors partly attributed this result to sample characteristics, noting that participating families were intact and relatively stable. Additionally, methodological factors, such as the small sample size, the laboratory setting, and participants’ awareness of being observed, may have further attenuated observed effects.

Seventeen studies considered the impact of both maternal and paternal AUD, whereas another 17 focused on the impact of paternal AUD. The five studies exclusively investigating maternal AUD with general parenting (Jacques et al., 2020, 2021, 2025; Rochat et al., 2019; Taber-Thomas & Knutson, 2021) are recent, suggesting a shift in the focus of the topic. Additionally, it is worth noting that the most recent study included in the present review (Seay, 2026), although including parents of both genders, was predominantly female, with mothers comprising 90.6% of the sample. As men usually have higher rates of AUD (Nolen-Hoeksema & Hilt, 2006), they are more easily recruited in research on AUD than women with AUD. By contrast, mothers traditionally assume the primary caregiver’s role in many societies and play an important role for children. This review, therefore, identifies a gap in the knowledge for the possible effects of maternal AUD and parenting practices.

#### 4.1.2. Alcohol-Specific Parenting

Only three studies focused on the associations between parental AUD and alcohol-specific parenting practices (Handley & Chassin, 2013; Van der Zwaluw et al., 2008; Ward & Snow, 2011) providing weak and inconsistent evidence for such an association. Indeed, no significant associations between parental AUD and parents’ alcohol supply to offspring were found (Ward & Snow, 2011). However, one study found that parents with AUD were more permissive toward offspring alcohol use and showed diminished alcohol-specific behavioral control, such as a lack of rules and monitoring (Van der Zwaluw et al., 2008). In particular, paternal (maternal) AUD was associated with less alcohol-specific behavioral control toward all (younger) adolescents (Van der Zwaluw et al., 2008). One study (Handley & Chassin, 2013) showed that mothers with high alcohol consumption and fathers with AUD more frequently disclosed negative experiences with alcohol, but only maternal AUD was associated with mothers having less legitimacy to regulate adolescent drinking.

Most of the literature on AUD and alcohol-specific parenting has studied offspring drinking as an outcome, which may have contributed to some of the inconsistent results. While employing strict rules on alcohol use and parents having alcohol-related communication with their children would likely reduce the risk of offspring drinking, one should consider the quality and frequency of these conversations and whether parental alcohol use might disrupt this path (Mares et al., 2011). The limited research considering AUD as an alcohol pattern, mostly studied with general parenting, might also help explain the inconsistent results.

### 4.2. Non-Dependent Parental Alcohol Use and Parenting

#### 4.2.1. General Parenting

Bryant et al. (2020) recently examined the impact of non-dependent parental drinking and found a significant positive association between parental alcohol consumption and children reporting experiencing negative outcomes. These adverse outcomes following their parents’ drinking reflected lower parent–child relationship quality and quantity, less attention, higher conflict, and more unpredictability.

Ten studies showed that non-dependent drinking, such as heavy drinking episodes, was associated with physical abuse, corporal punishment, and harsher or more demanding parenting, but the strength of the relationship varied by parental gender and drinking pattern (Edwards et al., 2009; Freisthler et al., 2023, 2020; Freisthler & Wolf, 2016; Keller et al., 2023; Kim et al., 2010; Lee et al., 2011; Spieker et al., 2001; Wolf & Freisthler, 2025; Wolf et al., 2021). Although such parental drinking patterns do not fulfill the diagnostic criteria for AUD, the acute effect of alcohol could affect parenting by, for example, reducing parental cognitive functioning (Weissenborn & Duka, 2003) and impairing parental supervision (Freisthler et al., 2014, 2020; Miller et al., 1997).

One study identified only an indirect effect of parental heavy drinking frequency on supervisory neglect, where the risk of negligent behaviors was higher, via increased depressive symptoms and low social support among heavy drinkers (Lloyd & Kepple, 2017). The lack of significance of this effect could be driven by their sample being derived from survey data on the general population, which is mostly characterized by light to moderate drinkers.

Although we cannot establish in all studies if the impaired parenting happened while parents were affected by alcohol, one experimental study (Lang et al., 1999) showed that compared with sober parents, acute alcohol intoxication caused parents to pay less attention to their children, provide inappropriate responses, and use more commands and off-task talk in adult–child interactions, threatening parental quality and efficacy. It is noteworthy that while the experiment compared the effects of intoxication between parents of children with and without behavioral problems, the results suggested that alcohol’s impact on parenting behavior was similar across both groups (Lang et al., 1999). Maternal care activities and mother–child relationship quality were also found to be higher among abstinent mothers than among mothers with moderate alcohol consumption (Tyrlík & Konecný, 2011).

The only experimental study involving non-dependent drinking (Lang et al., 1999) differed markedly from the AUD-focused studies. In this study, parents were randomly assigned to beverage conditions and child-behavior scripts, and interacted with trained child confederates rather than their own children. These confederates were scripted to display specific deviant or normative behaviors in order to examine parents’ reactions. While this design is not without ethical concern in line with the AUD-focused experimental studies, we consider the associated risks to be comparatively lower due to the role-play nature of the parent–child interactions.

Research seems to indicate that non-dependent alcohol use is generally associated with a reduction in positive parenting behaviors and quality, particularly when consumption reaches hazardous or higher levels (Amundsen et al., 2025; Freisthler & Wolf, 2023; Laslett et al., 2022). Higher alcohol consumption was linked to decreased supportive behaviors, such as comfort and encouragement (Amundsen et al., 2025), or praising and giving attention to their children (Freisthler & Wolf, 2023). A meta-analysis by Laslett et al. (2022) across five Asia-Pacific countries found that fathers’ heavy episodic drinking (HED) was associated with reduced positive parenting involvement, such as playing with their children, discussing personal matters, or assisting with homework. However, this association was strong and statistically significant only in Cambodia and Papua New Guinea, whereas it was not statistically significant in Indonesia and no evidence of an association was observed in China or Sri Lanka (Laslett et al., 2022). These cross-country differences may reflect samples with limited statistical power, cultural variation or differing HED prevalence rates.

A study highlighted that the impact of alcohol on parenting can be context-specific (Freisthler et al., 2023). Parents who drank on Super Bowl were significantly more likely to use aggressive discipline and punitive parenting, whereas parents drinking on Valentine’s Day were less likely to use aggressive discipline. These results suggest that the association between alcohol use and parenting can be influenced by the cultural norms of the occasion. In the same study, alcohol use was not significantly related to positive or nonpunitive parenting behaviors on either occasion.

The association between parental non-dependent alcohol use and the closeness of child–parent relationships varied by parental gender. In one study, maternal drinking had no significant effect on parent–child relationships, whereas paternal drinking significantly and negatively affected paternal closeness in another (Zhang et al., 1999). In general, men are considered to drink more alcohol, more often, and more problematically than women (Erol & Karpyak, 2015). Hence, gender differences in alcohol drinking patterns might partly explain gender differences in the impact of non-dependent parental alcohol use on parenting practices. However, these gender differences in drinking behaviors are gradually dissipating following social and cultural changes, and some studies found that women can be at an even higher risk of developing health and behavior problems when they drink than men (Erol & Karpyak, 2015).

Overall, some of the studies failed to find a strong association between light or moderate non-dependent parental alcohol use and parenting behaviors such as physical abuse and supervisory neglect. However, few studies examined the possible effects of light and moderate parental alcohol use on general parenting practices. Moreover, the concept of moderate drinking can be ambiguously defined and less concrete, making it harder to identify and establish effective measurement tools. Understanding this concept may also be limited for ethical reasons. Furthermore, moderate drinking episodes are usually situational and time-specific events during which parenting practices are rarely measured and studied. However, studying the effects of alcohol use on parenting behavior by parents affected by alcohol could help explain the extent to which such drinking patterns impair the ability to fulfill parental responsibilities.

#### 4.2.2. Alcohol-Specific Parenting

Parental practices promoting alcohol consumption such as children seeing their parents drinking, parents helping them buy alcohol, hearing positive arguments promoting alcohol use, and being permitted or encouraged to drink, increased as the frequency of both paternal and maternal drinking rose (Au et al., 2014, 2015; Maggs & Staff, 2018; Roberts et al., 2010; Smyth et al., 2010). However, some of these practices varied with parental gender. Two studies indicated that parental alcohol use was associated with more parental communication about alcohol (Mares et al., 2011) and less strict alcohol-specific rules (Van der Vorst et al., 2006). The results of another study showed that regular drinkers were significantly more tolerant toward introducing alcohol to adolescents at home than infrequent and non-drinking parents (Smyth et al., 2010).

While abstainers were less likely than heavy drinkers to allow their children to drink, few differences were identified between moderate/more infrequent drinkers and more frequent/heavier drinking parents (Maggs & Staff, 2018). Moreover, while mothers who consumed alcohol more often introduced their children to alcohol use, their initiation intentions were similar regardless of their level of alcohol use (Roberts et al., 2010), indicating that mothers’ norms regarding their drinking patterns minimally affect how they plan to transmit alcohol norms to their children.

Maggs et al. (2021) related research conducted during the recent COVID-19 stay-at-home pandemic period with parents that started to allow adolescent drinking with the family when it was not permitted previously. They found that having light and heavy-drinking parents increased the likelihood that children were newly permitted to drink. Fathers were also more likely to give permission. The authors noted that during the pandemic period, nearly one in six US parents allowed younger adolescents to drink at home for the first time (Maggs et al., 2021), which illustrates how the context can influence parenting behaviors. Given the lack of clear guidelines and inconsistent research results, it is understandable that even well-informed and -intentioned parents, as surely most are, may have difficulty in deciding the most beneficial approach. Additional knowledge-based information with useful implications for practice is thus necessary for parents.

Globally, alcohol is the most consumed drug. While most excessive drinkers in the population are not alcohol dependent (Bryant et al., 2020; Danielsson et al., 2012; O’Dwyer et al., 2019), there is no safe level of alcohol drinking according to recent breakthrough studies (Topiwala et al., 2022), with health risks increasing with consumption. Moderate drinking is more associated with adverse outcomes to vital organs (e.g., brain, heart) than abstinence. Indeed, low and moderate drinkers, who are more numerous than dependent drinkers, account for most alcohol-related harm, which seems to happen mostly in periods of acute intoxication (Danielsson et al., 2012; O’Dwyer et al., 2019). Research has shown that not only AUD but also non-dependent drinking patterns can impair cognitive function and the performance of everyday tasks (Gunn et al., 2018; Weissenborn & Duka, 2003). Hence, even low quantities of alcohol consumption can confer risks to parenting behaviors.

Although the consequences remain understudied, it is plausible to assume that the intoxicating effect of alcohol may reduce parental capacity independent of whether parents have AUD or are drinking non-dependently or heavily episodically.

### 4.3. Strengths and Limitations

This review identified 68 studies, published from 1991 to 2026, which constituted a solid base of relevant research on the associations between parental alcohol use and parenting practices. We conducted a clear and rigorous systematic review process, following the PRISMA methodology, with Prospero registration and a comprehensive search strategy. Different alcohol use patterns and a wide range of parenting factors were included using a previously studied categorization of variables.

There are, however, some limitations. Many of the included studies had cross-sectional designs, which limited their ability to draw causal inferences from their observational data. Further, 18 of the 28 studies examining the associations between non-dependent parental alcohol use and parenting practices were cross-sectional. In a cross-sectional design, the directionality of the relationship between a parenting practice and alcohol consumed by that parent remains uncertain and should be studied using a prospective design. On the contrary, 22 of the 40 studies examining the associations between parental AUD and parenting practices had a longitudinal design, which may allow insights into the causal relationships.

Further limitations are that the strength of the reported relationships could not be determined and that some included papers were based on the same sample, perhaps reducing the empirical base for the studies. Inconsistency in the use of concepts as well as in the definitions of parental alcohol use and parenting practices were also identified in the review. The terminology used in the literature frequently overlaps, standardized measures across studies are lacking, and the analysis approaches vary. Comparisons between studies are therefore limited and hindered by data heterogeneity. This review also found that more moderate drinking patterns and parenting relations remained understudied and therefore less understood. Most of the results related to non-dependent alcohol patterns were reported as binge drinking, heavy drinking, and intoxication. Evidence regarding the statistical significance of this pattern remains unclear, possibly due to the small number of studies and the reliance on cross-sectional designs.

Research on alcohol-specific parenting practices is relatively limited and largely based on a small cluster of studies from the Netherlands, primarily from the same research group, highlighting the need for broader empirical contributions across contexts.

Whether parenting behavior was measured while parents were intoxicated by alcohol is not known in most of the studies, making it impossible to say that the chemical effect causes impaired parenting, although the results of experimental studies seem to support this. Other underlying factors related to lifestyle, mental health, sociodemographic factors (e.g., age, gender, family, and social context), and socioeconomic status may impact both parental alcohol use and parenting. Disregarding the broader environment around the parenting context could thus lead to inconsistent or flawed results (Koning et al., 2012).

Important limitations should be acknowledged when interpreting the findings of this review. First, publication bias cannot be ruled out, as studies reporting significant or adverse associations between parental alcohol use and parenting practices may be more likely to be published than studies with null or inconclusive findings. Although a formal assessment of publication bias was not feasible given the heterogeneity of study designs, outcomes, and measures, this bias may have contributed to an overrepresentation of negative associations in the synthesized evidence.

Second, and more critically, the evidence base is characterized by a pronounced geographical bias. The vast majority of included studies were conducted in the United States (53 of 68), with comparatively few from Europe, Australia, Asia, and only one from Africa. This strong concentration in Western countries reflects a broader tendency in psychological and public health research to rely on samples from Western, Educated, Industrialized, Rich, and Democratic (WEIRD) societies (Henrich et al., 2010). Such sampling substantially limits the generalizability of the findings, particularly for alcohol-specific parenting practices (e.g., rule-setting, communication about alcohol, monitoring of drinking), which are highly sensitive to cultural norms, drinking contexts, family structures, and social expectations. Parenting norms, meanings attached to alcohol use, and the acceptability of drinking within family life vary across cultural settings, as do the measurement tools used to assess both alcohol consumption and parenting behaviors. Consequently, findings derived largely from WEIRD contexts may fail to capture the full variability of how parental drinking relates to parenting in non-Western or Global South settings and risk ethnocentric interpretations of these associations. Future research would benefit from more geographically and culturally diverse study designs to strengthen external validity and provide a more globally representative understanding of the relationship between parental alcohol use and parenting practices. In addition, we only included English-language studies, which may have excluded relevant papers and contributed to the biased findings.

### 4.4. Key Contributions and Implications

The impact of parental alcohol use on parenting quality has been researched over several decades. However, the majority of studies have focused on clinical populations of parents with AUD. This review expands the existing body of knowledge by providing a critical synthesis of research on an understudied subtopic within this broader field: the influence of non-dependent parental alcohol use on parenting behaviors and outcomes. It moves beyond the conventional clinical view of AUD, to shed some light on the often-overlooked consequences of moderate drinking patterns, revealing a more nuanced and, probably, concerning reality for many families.

One of the review’s most significant contributions is shifting the focus beyond AUD toward a spectrum of drinking behaviors, extending the lens from clinical populations to everyday life and the often-overlooked consequences of moderate drinking. This shift is crucial, as non-dependent drinking is more prevalent than AUD and affects a substantially larger number of families, constituting an important public health concern. By broadening the population of interest, the review expands understanding of the potential risks associated with parental alcohol use and highlights the need for preventive measures beyond clinical settings. It also exposes hidden risks, as the normalization of moderate drinking, particularly in social contexts, can mask its potential consequences. The effects of non-dependent drinking on parenting may be subtle and gradual, making them harder to detect than the more visible consequences of AUD. This challenges assumptions that only clinical drinking problems harm parenting and child well-being, with important implications for public health messaging and prevailing notions of “safe” drinking levels for parents. Overall, the shift underscores a critical gap in knowledge regarding how moderate drinking patterns affect parenting, calling for new theoretical frameworks and robust research designs to clarify these relationships in family life.

An additional contribution from this review is the potential insight into the complex factors that may influence the relationship between parental alcohol use and parenting practices. Although the review was not explicitly focused on these factors, the included studies suggest possible nuances that can shape this relationship outcome. Some results appear to indicate that gender could play a differentiating role, with mothers’ and fathers’ drinking behaviors possibly affecting parenting in distinct ways. This interpretation raises questions about the assumption of a uniform impact and points to the need for further investigation into gender-specific interventions and support systems. Furthermore, the review hints that parenting may be influenced by external contextual factors, such as financial strain, unemployment, or even global crises like pandemics, which might exacerbate the effects of parental alcohol use. The review suggests that a more holistic approach, addressing the wider social and economic context, could be beneficial in developing effective interventions. 

Importantly, this review highlights that, despite the high prevalence of non-dependent parental drinking, the current evidence base does not yet support a single, clear conclusion regarding its impact on parenting practices. Making this gap visible constitutes a key contribution from the review and underscores the need for further research in this area.

## 5. Conclusions

The findings of this systematic review indicate that parental alcohol use is frequently associated with poorer quality parenting practices. However, the strength, direction, and consistency of these associations vary across drinking patterns, parenting domains, and study contexts. In particular, while alcohol use disorders (AUD) have been more consistently linked to impairments in general parenting practices, findings related to non-dependent alcohol use and alcohol-specific parenting remain more mixed and less conclusive. This variability highlights the need for caution in drawing overarching conclusions and underscores important gaps in the current evidence base.

Several limitations in the literature were identified. Few studies examine alcohol-specific parenting practices in the context of AUD, despite AUD being the most extensively studied drinking pattern in relation to general parenting. Conversely, alcohol-specific practices are more often examined in studies of non-dependent drinking, although this body of evidence remains limited in scope and methodological consistency. In addition, maternal AUD and gender-specific associations between parental drinking and parenting practices remain underrepresented, despite indications that associations may differ between mothers and fathers. Light to moderate drinking patterns, the most prevalent forms of alcohol consumption in the general population, also remain ambiguously defined and comparatively understudied, limiting understanding of their potential implications for parenting.

Parenting is a complex, multidimensional, and dynamic construct shaped by reciprocal psychological, biological, social, and cultural processes, as well as by broader contextual influences such as socioeconomic conditions and gender norms. Consequently, parental alcohol use should not be examined in isolation but rather situated within the wider ecological context in which parenting occurs. The predominance of studies conducted in Western countries further constrains the generalizability of current findings and highlights the need for more geographically and sociocultural diverse research.

Overall, this review underscores critical gaps in knowledge regarding how different patterns of parental alcohol use relate to parenting practices, particularly in everyday, non-clinical contexts. Addressing these gaps through robust longitudinal designs and more diverse samples is essential for clarifying pathways of influence and informing prevention efforts and parenting interventions. Improved understanding of modifiable risk and protective factors may ultimately support more effective alcohol-related and parenting-focused public health strategies.

## Figures and Tables

**Table 1:** (A). Description of the included studies: Alcohol use disorder AUD as exposure. (B) Description of the included studies: Non-dependent alcohol use as exposure.

Author (Year) Country	*N*	Study Design, Sample Type	Age of Offspring	Exposure Measure	Outcome Measure	Main Findings
(A)						
Anda et al. (2002) USA	9346 adults	Retrospective cohort	0 to 18 years (reported retrospectively)	Parental alcohol abuse (alcoholism)	Abuse (emotional, physical, and sexual) ^GP^	Parental alcohol abuse increased the risk of abuse (emotional abuse AOR = 2.9, 95%CI = 2.3–3.6; physical abuse AOR = 1.9, 95%CI = 1.6–2.3; sexual abuse AOR = 1.9, 95%CI = 1.6–2.4).
Bijttebier and Goethals (2006) Belgium	207 children	Cross-sectional	10 to 14 years	Parental AUD	Family environment ^GP^	Compared with non-alcoholic families, parental alcohol problems were associated with lower family cohesion and poorer family organization.
Chassin et al. (1996) USA	454 adolescents	Longitudinal, 3 waves (annual)	10.5 to 15.5 years	Alcohol abuse or dependence	Monitoring ^GP^	Paternal alcoholism was related to less paternal monitoring, while maternal alcoholism was not associated with maternal monitoring.
Chassin et al. (1993) USA	454 adolescents	Cross-sectional	10.5 to 15.5 years	Alcohol abuse or dependence	Monitoring ^GP^	Alcoholic parents were less likely to monitor adolescent activities. Decline in parental monitoring was related to clinical alcoholism over the lifetime rather than parents’ current alcohol consumption.
Edwards et al. (2004) USA	217 families: 217 children, 217 mothers and 208 fathers	Longitudinal, two waves (12 m, 18 m)	12 to 18 months	Families with an alcoholic father; alcohol abuse and dependence	Parent–infant attachment ^GP^	Higher maternal alcohol symptoms were associated with stable insecure mother–infant attachment relationships compared with those who were secure. Similar effects were not found for the father–infant attachment relationship.
Eiden et al. (1999) USA	204 families.	Cross-sectional	1 year	Families with alcoholic fathers; alcohol abuse and dependence diagnoses for current alcohol problems.	Parent–infant interaction (negative affect, positive affect, or involvement, sensitivity) ^GP^	Fathers’ alcoholism was associated with more negative father–infant interactions indicated by lower paternal sensitivity, positive affect and verbalizations, and higher negative affect. Mothers with higher current alcohol problems were less sensitive in their interactions with infants.
Eiden et al. (2009a) USA	227 families	Longitudinal, 6 waves (12, 18, 24, 36, 48 months + kindergarten)	1 to 2 years	Alcohol abuse and dependence: fathers’ alcoholism; both parents alcoholic.	Parental sensitivity and warmth ^GP^	Fathers’ alcohol diagnosis at 12–18 months was associated with lower paternal warmth/sensitivity at 2 years.
Eiden et al. (2007) USA	227 families	Longitudinal, 6 waves (12, 18, 24, 36, 48 months, kindergarten)	1 to 2 years	Alcohol abuse and dependence: father’s alcoholism; both parents alcoholic.	Parenting quality: sensitivity/warmth during parent–child interaction ^GP^	Fathers’ alcohol diagnosis when children were 12–18 months was associated with lower paternal warmth and sensitivity at 2 years.
Eiden et al. (2004a) USA	226 families	Longitudinal, 4 waves (12, 24, 36, 60 months)	1 to 3 years	Alcohol abuse and dependence: fathers’ alcoholism.	Parental warmth ^GP^	Alcoholic fathers displayed lower warmth toward their offspring than non-alcoholic fathers
Eiden et al. (2002) USA	223 families	Cross-sectional	1 year	Alcohol abuse and dependence: father’s alcoholism; both parents with alcohol problems.	Parental sensitivity during parent–child interaction ^GP^	Both maternal and paternal alcohol problems were associated with lower sensitivity (higher negative affect, lower positive engagement, and lower sensitive responding) during parental–infant interactions.
Eiden and Leonard (2000) USA	216 families	Cross-sectional	1 year	Alcohol abuse and dependence: fathers’ alcoholism.	Paternal aggravation and warmth ^GP^	Fathers’ alcoholism was associated with higher paternal aggravation with the infant.
Eiden et al. (2004b) USA	222 families	Longitudinal, 3 waves (12, 18, 24 months)	1 to 2 years	Alcohol abuse and dependence: father’s alcoholism.	Parental sensitivity during parent–child interactions ^GP^	Higher paternal alcohol consumption at 1 year was associated with negative and less sensitive behavior at 2 years.
Eiden et al. (2009b) USA	213 families	Longitudinal, 6 waves (12, 18, 24, 36 months, kindergarten, fourth grade)	1 to 3 years	Alcohol abuse and dependence: Fathers’ alcoholism.	Marital aggression ^GP^	Fathers’ alcohol diagnosis was associated with higher levels of marital aggression.
Elam et al. (2020) USA	825 parents (457 mothers and 368 fathers)	Longitudinal, 2 waves (6 years apart)	10 to 14 years at T1	Parental AUD.	Parents’ knowledge of child activities and interests ^GP^	Mothers’ AUD was associated with less knowledge of their children (β = −0.14, SE β = 0.05). No association was detected between fathers’ AUD and knowledge.
Famularo et al. (1992) USA	190 cases from juvenile court involving the maltreatment of children	Cross-sectional	Children (age not indicated but under 18 years)	Parental alcohol misuse.	Parental physical and sexual maltreatment ^GP^	Parental alcohol misuse was significantly associated with child physical maltreatment but not with sexual maltreatment.
Finan et al. (2015) USA	492 adolescents	Longitudinal, 2 waves (annual)	14 to 19 years	Parental problem drinking.	Parent–adolescent communication and family cohesion/closeness ^GP^	Maternal problem drinking negatively influenced daughter–mother communication (β = −0.09, *p* < 0.05) and paternal problem drinking negatively influenced daughter–father communication (β = −0.13, *p* < 0.01). Only paternal problem drinking was negatively related to son–father communication (β = −0.21, *p* < 0.001). These results suggest less openness and problematic communication. For both boys and girls, mother and father problem drinking were associated with lower family cohesion.
Finger et al. (2010) USA	183 families	Longitudinal, 5 waves (12 m, 18 m, 24 m, 36 m; kindergarten)	1 to 5 years	Alcohol abuse and dependence: fathers’ alcoholism.	Paternal harsh parenting: negative affect, high control, and overreactive discipline ^GP^	Fathers’ alcohol diagnosis predicted marital aggression. It was suggested that marital aggression can be one mechanism linking fathers’ alcohol diagnosis to harsh parenting.
Handley and Chassin (2013) USA	472 adolescents, 268 mother, 204 fathers	Longitudinal, 2 waves (6; 4-year follow-up)	12.6 (SD = 1.7) years on average	Parental AUD (lifetime and past year diagnoses). Parental alcohol use: frequency and quantity in the past year.	Alcohol-specific parenting. General parenting: social support and consistency of discipline ^GP; AP^	Maternal current AUD was associated with less social support to their adolescents (*B* = 0.21, *p* < 0.05) and they were viewed as having less legitimacy to regulate adolescent drinking (*B* = 0.09, *p* < 0.05). Higher levels of maternal drinking predicted more frequent maternal disclosure of negative experiences with alcohol (*B* = 0.46, *p* < 0.05) and less consistency of discipline (*B* = −0.20, *p* < 0.05). Among fathers, more alcohol disclosure was the only parenting behavior affected by fathers’ AUD.
Jacob et al. (1991) USA	121 families	Experimental (cross-sectional)	Adolescent (average age for the alcoholic group = 13.4)	Fathers’ alcoholism, alcohol consumption by parents during the experiment.	Parent–child interactions ^GP^	Alcohol use on site had no general effect on father–child, mother–child dyad, or father–mother–child interactions. However, fathers’ negative behavior was significantly more affected by the drinking condition than that of mothers. Mothers decreased and fathers and children increased their problem solving from no-drinking to drinking sessions. Fathers’ alcoholism showed no effects on parent–child interaction.
Jacob et al. (2001) USA	100 families	Experimental (cross-sectional)	10 to 18 years	Fathers’ alcoholism: high and low antisocial alcoholism. Alcohol consumption during one of two sessions of the experiment.	Family interactions ^GP^	Family interactions were related to both fathers’ alcoholism type and alcohol consumption. Alcohol consumption increased negative family interactions, particularly with high antisocial alcoholic fathers.
Jacques et al. (2025) USA	201 mother-child dyads	Longitudinal, 3 waves (each one year apart)	Approximately, from wave 1, 26 months old; to wave 3, 48 months old.	Maternal alcohol dependence	Maternal insensitivity to children’s emotional distress (e.g., ignoring, yelling, or failing to provide comfort when the child expresses sadness or fear). ^GP^	Maternal alcohol dependence symptoms at Wave 1 predicted significant increases in mothers’ insensitivity to their children’s emotional distress over the following year.
Jacques et al. (2021) USA	201 mothers and their children	Longitudinal, 3 waves (every ~2 years)	2 years at wave 1	Maternal alcohol dependence.	Maternal insensitivity, disengagement, and intrusiveness to child distress ^GP^	Higher levels of maternal alcohol dependence predicted more disengagement from children’s distress one year later. Maternal alcohol dependence symptoms were not associated with mothers’ intrusive responses to distress.
Jacques et al. (2020) USA	201 mothers and their children	Longitudinal, 2 waves (T1 child age~2; T2 child age~3)	2 years at wave 1	Maternal alcohol dependence.	Maternal harsh caregiving ^GP^	Harsh parenting among alcohol-dependent mothers increased over time in a more stressful discipline context, but not in a parent–child play context.
Kachadourian et al. (2009) USA	197 families	Longitudinal, 3 waves (12; 24; 36 months)	1 to 3 years	Paternal alcoholism.	Warmth and sensitivity ^GP^	Paternal alcohol diagnosis at 1 year was predictive of lower warmth/sensitivity at 2 years (β = −0.34, *p* < 0.05), but higher at 3 years (β = 0.22, *p* < 0.05).
Keller et al. (2024) USA	337 parents	Cross-sectional	5 to 12 years	Parental problem drinking 16.6% met the criteria for AUD	Supportive Reactions (problem-focused responses, emotion-focused responses, and encouragement of emotional expression) Nonsupportive Reactions: (Distress reactions, minimizing the child’s feelings, and punitive responses) ^GP^	There was a significant direct association between Parental Problem Drinking (PPD) and nonsupportive parenting behaviors. Instead, the relationship between PPD and supportive parenting was only indirect, mediated through parent depression.
Keller et al. (2021) USA	377 college students	Cross-sectional	Childhood (retrospectively reported)	Parental problem drinking.	Parental reactions to children’s negative emotions ^GP^	Parental problem drinking by both mothers and fathers was associated with greater same-parent minimization, distress, and punitive reactions, and to less problem-focused, emotion-focused, and encouragement of the expression of negative emotions.
Kelley et al. (2011) USA	388 college students	Cross-sectional	0 to 16 years (retrospectively reported)	Maternal and paternal alcoholism.	Parent–child relationship ^GP^	Negative parent–child relationship with greater alienation and emotional longing, poorer communication, and less trust was associated with parental alcohol abuse. Both maternal and paternal alcoholism was associated with more negative mother–child and father–child relationships, respectively.
Moser and Jacob (1997) USA	137 families	Experimental (cross-sectional)	10 to 17 years	Parental alcoholism. Alcoholic beverages were made available to the parents during sessions.	Parent–child interactions ^GP^	Alcoholic families exhibited more impaired parent–child interactions and lower positivity than control groups. The dual and mother-only alcoholic families had the highest negativity and impaired interactions. Families with at least an alcoholic mother had the strongest impact on parent–child interactions, both for negative and for positive behavior, than the father-only alcoholic and normal control families. This is suggested to be a protective effect of having a non-alcoholic mother.
Ohannessian (2012) USA	683 adolescents	Longitudinal, 2 waves (annual)	15 to 17 years	Parental problem drinking.	Adolescent–parent communication ^GP^	Both maternal and paternal problem drinking was associated with more problematic, less open adolescent–parent communication. For boys, paternal problem drinking negatively predicted adolescent–father communication (β = −0.18, *p* < 0.01). However, maternal problem drinking was not significant. For girls, both maternal and paternal problem drinking negatively predicted adolescent–mother communication (β = −0.15, *p* < 0.01) and adolescent–father communication (β = −0.14, *p* < 0.01), respectively.
Rangarajan (2008) USA	515 adults	Cross-sectional	0 to 16 years (reported retrospectively)	Parental alcoholism.	Parental attachment and family stressors ^GP^	Paternal alcoholism, but not maternal alcoholism, had a negative association with parental attachment security. Family stressors partially mediated the effects of paternal alcoholism on paternal attachment. The results showed support for the detrimental effects of paternal alcoholism (β = −0.38, *p* < 0.01) on paternal attachment.
Rochat et al. (2019) South Africa	1505 mothers or caregivers with 1536 children	Cross-sectional	7 to 11 years	Maternal AUD: hazardous drinking.	Parenting stress index: parental distress, parent–child relationship dysfunction, and finding the child difficult to parent ^GP^	Mothers in the hazardous drinking group were associated with higher parental distress, parent–child relationship dysfunction, and their child being difficult to parent than non-drinkers and mothers who drink less.
Rutherford et al. (1998) USA	173 adult men	Cross-sectional	0 to 16 (reported retrospectively)	Familial history of alcoholism (fathers).	Parental care and protection ^GP^	The group with a family history of paternal alcoholism reported their fathers being less caring than the non-alcoholic family group.
Schacht et al. (2009) USA	235 families	Longitudinal, 3 waves (annual)	5 to 6 years	Fathers’ problem drinking.	Paternal positive parenting and marital conflict ^GP^	Paternal problem drinking was associated with paternal negative marital conflict and decreased positive parenting.
Seay (2026) USA	1116 families: parent or caregiver (90.6% female) and child aged 8 and older. Sample was randomly split into two. *N* = 520 families. For parental monitoring with children aged 10 or older: *n* = 383 families.	Longitudinal, 2 waves (wave 1—February 2008 to April 2009; wave 2—18 months later)	8 to 17.5 years Measures for exposure to violence and trauma were collected from children 8 years of age and older, while the measure for parental monitoring was restricted to children 10 years of age and older.	Parental problematic alcohol use	Parental monitoring; Exposure to Violence involving adults in the home; Harsh discipline (e.g., physical assault or aggressive discipline techniques); Emotional maltreatment. ^GP^	Higher levels of problematic alcohol use were associated with increased exposure to violence at home and more parental monitoring. There were no significant mediating pathways found for harsh discipline or emotional maltreatment in relation to parental alcohol use.
Senchak et al. (1995) USA	244 college students	Cross-sectional	Childhood (early family environment reported retrospectively)	Fathers’ alcoholism.	Father warmth and parental conflict ^GP^	Children of alcoholics reported less father warmth than children of divorce (M = 2.07) or controls (M = 2.15). They also reported greater parental conflict (M = 2.36) than controls (M = 2.17).
Su et al. (2018) USA	1282 adolescents and parents	Longitudinal, 2 waves (~2-year apart)	12 to 17 years	Parental alcohol dependence symptoms.	Positive parenting (parental involvement, communication, and closeness) ^GP^	Fathers’ alcohol dependence symptoms were negatively associated with fathers’ positive parenting behaviors (β = −0.16, *p* < 0.01), whereas mothers’ alcohol dependence symptoms were not significant.
Taber-Thomas and Knutson (2021) USA	311 mothers	Longitudinal, 2 waves (~3 years apart)	4 to 8 years in the first year	Maternal history of alcohol use.	Harsh punitive and inconsistent discipline, supervisory and care neglect. ^GP^	Maternal history of alcohol use was associated with more inconsistent discipline in the first year and higher levels of subsequent supervisory neglect in the second year. Harsh parenting was not significant.
Tweed and Ryff (1996) USA	193 college students	Cross-sectional	0 to 18 (reported retrospectively)	Paternal alcoholism.	Family climate, family climate under stress, and parent–child relationships ^GP^	Respondents with alcoholic fathers described a more negative family climate with higher levels of conflict and lower cohesion and expressiveness than those with non-alcoholic families. Adult children of alcoholics described more negative relationships with their alcoholic fathers.
Van der Zwaluw et al. (2008) The Netherlands	428 families with two adolescents	Longitudinal, 3 waves (annual)	Mean age at T1: 15.2 years (SD = 0.60) for older adolescents, 13.4 years (SD = 0.50) for younger adolescents	Parents’ problem drinking.	General parenting: parental behavioral control and parental support. Alcohol-specific parenting: parents allowing children to drink and behavioral control regarding their children’s alcohol consumption ^GP; AP^	Parental problem drinking was in general not associated with parenting with some exceptions. Alcohol-specific parenting: the problem drinking of both fathers and mothers at T1 was positively related to permissiveness toward older adolescents at T2. Parental problem drinking at T2 on alcohol-specific behavioral control at T3 was significant for the father for both adolescents and for the mother for the younger adolescent. General parenting: only maternal problem drinking at T2 showed a significant association with behavioral control toward the youngest adolescent at T3 (b = −0.12, *p* < 0.01).
Ward and Snow (2011) Australia	388 parents	Cross-sectional	14 to 16 years	Parental AUD.	Parental supply of alcohol ^AP^	Alcohol supply to adolescents was not significantly associated with parents’ AUD.
(B)						
Amundsen et al. (2025) Norway	4260 parents (2524 mothers and 1736 fathers)	Cross-sectional	4 to 12 years	Parental alcohol use	Supportive and non-supportive emotion socialization behaviors (ESBs) ^GP^	Higher levels of parental alcohol use were significantly associated with decreased supportive ESBs (e.g., encouragement, empathy) and increased non-supportive ESBs (e.g., minimization, punitive reactions). Only the high-risk alcohol group showed significantly lower supportive ESBs compared to the no-risk/abstainer group.
Au et al. (2014) Hong Kong—China	1757 adolescents	Cross-sectional	14.7 ± 2.0 years	Parental drinking (frequency).	Nine parental pro-drinking practices: seeing parents (a) drink and (b) drunk; heard parents mention (a) the benefits of drinking and (b) alcohol tasted good; helped parents (a) buy alcohol, (b) open bottle and (c) pour alcohol; and parents (a) encouraged to drink and (b) trained drinking capacity ^AP^	The prevalence of the parental pro-drinking practices was low in non-drinking parents but increased dramatically with the parental drinking frequency and number of drinking parents.
Au et al. (2015) Hong Kong—China	1087 adolescents	Cross-sectional	14.6 ± 2.0 years	Parental drinking (frequency).	Nine parental pro-drinking practices—see Au et al. (2015) ^AP^	The parental pro-drinking practices increased overall with both paternal and maternal drinking frequencies.
Bryant et al. (2020) UK	997 families (one parent and child)	Cross-sectional	10 to 17 years	Parental alcohol use and children’s exposure.	Negative general parenting outcomes from their parents’ drinking (e.g., parent–child relation, conflict, and attention) ^GP^	Higher levels of parental alcohol consumption were significantly associated with an increased likelihood children reported experiencing negative outcomes. Children having seen their parent tipsy or drunk were also more likely to report negative outcomes.
Edwards et al. (2009) USA	226 families	Longitudinal, 5 waves (18, 24, 36, 48, 60 months)	1 to 5 years	Binge drinking, alcohol quantity and frequency.	Discipline: laxness and overactivity ^GP^	Paternal binge drinking was associated with paternal overactivity, suggesting harsher and more demanding parenting. Maternal binge drinking was not associated with discipline.
Freisthler et al. (2014) USA	3023 parents	Cross-sectional	12 years or younger	Alcohol use categories: lifetime abstainers, ex-drinkers, light drinkers, moderate drinkers, infrequent heavy drinkers, occasional heavy drinkers, and frequent heavy drinkers.	Supervisory neglect ^GP^	Alcohol use was not associated with supervisory neglect, except for frequent heavy drinking that was significantly associated with leaving a child in a place where safety was unknown.
Freisthler et al. (2023) USA	255 parents (Super Bowl) and 184 parents (Valentine’s day)	Longitudinal—Ecological Momentary Assessments (EMA); 14 days, 3 daily assessments. Wave 2.	2 to 12 years	Drinking frequency. During the 14-day assessment, on days 7 and 14, to recall their drinking for the past week and the time frames	Aggressive discipline (e.g., psychological aggression and corporal punishment) Punitive parenting (disciplinary actions) Nonpunitive parenting (non-harsh disciplinary techniques) Positive parenting (e.g., giving a child full attention) ^GP^	The impact of alcohol on parenting was context-specific: Super Bowl: parents who reported drinking were more likely to use aggressive discipline (OR = 2.560) and punitive parenting (OR = 2.701) during consumption period. Valentine’s Day: parents who reported drinking were less likely to use aggressive discipline (OR = 0.197). Alcohol use was not significantly related to positive or nonpunitive parenting behaviors on either occasion.
Freisthler and Wolf (2023) USA	302 parents	Longitudinal—Ecological Momentary Assessments (EMA); 14 days; second wave.	2 to 12 years	Drinking frequency. Baseline Survey: previous 12 months; EMA Reports: During the 14-day assessment, on days 7 and 14, to recall their drinking for the past week—days and the time frames	Punitive parenting (e.g., corporal punishment, psychological aggression, and deprivation of privilege) Non-punitive parenting (e.g., explaining/teaching, rewards, and monitoring); Positive parenting (e.g., praising, giving attention). ^GP^	Parents reported less nonpunitive parenting during the same time periods in which they were drinking. Alcohol use was associated with lower odds of positive parenting in the subsequent time period. The study did not find a significant relationship between alcohol use and punitive parenting
Freisthler and Wolf (2016) USA	3023 parents	Cross-sectional	12 years or younger	Alcohol use categories: lifetime abstainers, ex-drinkers, light drinkers, moderate drinkers, and heavy drinkers.	Physical abuse ^GP^	Ex-drinkers, light drinkers, and heavy drinkers used physical abuse more often than lifetime abstainers. Moderate drinking was not related to physical abuse.
Freisthler et al. (2020) USA	1599 parents	Cross-sectional (mixed methods)	10 years or younger	Parental alcohol use frequency.	Lack of supervision and physical harm ^GP^	One in four parents reported that their own drinking caused them to not supervise their child closely enough, whereas one in eight stated that it caused them to physically harm their child. Parents with higher continued volumes of drinking were less likely to say that drinking caused them to not supervise their child closely enough.
Freisthler et al. (2015) USA	2152 parents	Cross-sectional	12 years or younger	Alcohol use: frequency and volume.	Supervisory neglect, physical neglect ^GP^	Unlike volume, the frequency of drinking was related to different types of supervisory neglect depending on the context. However, it was not related to physical neglect. Different social mechanisms may underlie the relation between drinking contexts and neglect.
Keller et al. (2023) USA	199 mother–father–child triads	Cross-sectional	6 to 12 years	Parental alcohol use	Harsh parenting: Rejection and invalidation; Coercion. ^GP^	Mother drinking was associated with mother harsh parenting. The findings seem to indicate that even subclinical levels of alcohol use can be a risk to parenting quality and child emotional development. In this study, paternal alcohol drinking was not significantly directly associated with the father’s own parenting behaviors.
Kim et al. (2010) USA	488 mothers	Longitudinal, 4 waves (birth; 1; 2; 3 years)	0 to 3 years	Maternal alcohol use: frequency and quantity.	Harsh parenting ^GP^	Maternal alcohol use had a strong significant direct effect on harsh parenting at 3 years.
Lang et al. (1999) USA	192 parents	Experimental (cross-sectional)	5- to 12-year-old boys	Alcohol intoxication during experimental session.	Adult–child interactions (parents were paired with a boy who role-played as their son) ^GP^	Parents under alcohol intoxication exhibited less attention and productive work and provided more commands, indulgences, and off-task talk in the adult–child interactions than sober parents.
Laslett et al. (2022) Cambodia; China; Indonesia; Papua New Guinea; and Sri Lanka.	4562 fathers from five low- and middle-income countries (LMIC) in the Asia-Pacific region.	Cross-sectional	Not indicated. Fathers aged 18–49 years.	Heavy Episodic Drinking (HED) 6 or more drinks on one occasion. Classification: Participants who reported engaging in HED versus non-HED drinkers and abstainers Frequency.	Fathering involvement: Playing with or participating in activities with their children. Discussing personal matters with their children. Helping children with homework ^GP^.	Fathers’ HED was significantly associated with less positive parental involvement overall. However, there are regional variations. The association was statistically significant in Cambodia and Papua New Guinea. Followed the same direction in Indonesia but it was not significant. No evident association in China or Sri Lanka.
Lee et al. (2011) USA	2309 fathers	Cross-sectional	3 years	Paternal alcohol use: no drinking, moderate drinking, and heavy drinking.	Corporal punishment ^GP^	Paternal heavy alcohol use was associated with fathers’ increased use of corporal punishment.
Lloyd and Kepple (2017) USA	2990 parents	Cross-sectional	0 to 12 years	Parental heavy drinking frequency.	Supervisory neglect ^GP^	Frequency of heavy drinking showed no significant direct effect on supervisory neglect, but an indirect effect was shown via depressive symptoms and decreased social support (*B* = 0.015, *p* < 0.01).
Maggs et al. (2021) USA	911 families with two adolescent siblings and one parent	Longitudinal, 2 waves (T1 pre-pandemic Mar 2019–Mar 2020; T2 during shutdown 1 May–15 June 2020)	At T1: Older M = 15.67 [SD = 0.68] years Younger M = 13.14 [SD = 1.11] years.	Parental alcohol use: abstainers, light drinkers, and heavy drinkers.	Parent allows adolescents to drink with the family ^AP^	Nearly one in six parents, who did not permit adolescent drinking before, allowed it during the COVID-19 lockdown. Adolescents who had light or heavy drinking parents were more likely to be newly permitted to drink. Permitting drinking was more likely among parents who were fathers (OR = 1.95) and light drinkers (vs. abstainers; OR = 2.02).
Maggs and Staff (2018) UK	10,210 families	Longitudinal, 2 waves (T1 pre-pandemic Mar 2019–Mar 2020; T2 during COVID-19 1 May–15 Jun 2020)	1 to 14 years	Parental alcohol use: frequency and quantity when child was 11 years, divided into categories from abstainer to frequent and heavier use.	Parent alcohol permissibility: allow drinking when the child was 14 years ^AP^	Abstainers were less likely than current heavy drinkers to allow their child to drink. However, moderate and more infrequent drinking parents were not less likely to allow offspring alcohol use than more frequent and heavier drinkers.
Mares et al. (2011) Netherlands	428 families	Longitudinal, 5 waves (from infancy to age 14, irregular intervals)	13 to 16 years (two adolescents at T1)	Parental alcohol consumption: frequency and intensity.	Alcohol-specific communication ^AP^	Only paternal alcohol use was related to less alcohol-specific communication from the father to older adolescents (β = −0.13, *p* < 0.05). Maternal alcohol use, with both adolescents, and paternal drinking with the youngest, were not significantly associated with their communication about alcohol.
Roberts et al. (2010) Australia	161 mothers	Cross-sectional	10 to 14 years	Mothers’ alcohol consumption: higher risk, acceptable risk, and non-drinkers.	Intention to introduce their adolescent to alcohol ^AP^	Mothers’ intentions to initiate their children into alcohol use were remarkably similar despite own use. While mothers’ favorable responses increased with their alcohol use, the differences found were largely between the non-drinking and acceptable risk groups, with only small or medium effects.
Smyth et al. (2010) Ireland	234 parents	Cross-sectional	13 to 17 years	Parental alcohol consumption.	Introducing children to alcohol at home and alcohol provision ^AP^	On introducing children to alcohol in the home, parents of teenagers who drank infrequently (OR = 2.6 [1.3, 5.2]) or not at all (OR = 10 [2.6, 37.9]) were significantly more likely to disagree with this idea. Parents who regularly drank demonstrated more permissive attitudes toward teenage drinking (M = 2.8) than infrequent (M = 1.7) and non-drinking parents (M = 1.4). Provision of alcohol was not associated with parental drinking.
Spieker et al. (2001) USA	185 adolescent mothers	Longitudinal, 2 waves (early postpartum period; age 5)	6 years	Mothers’ alcohol use (quantity and frequency).	Unrealistic expectations, negative attributions, coercive or negative control of children’s behavior, and impaired mother–child interaction ^GP^	Mothers’ alcohol use was only significantly correlated with maternal reports of negative control (r = 0.22, *p* < 0.01) such as yelling, pushing, and spanking. Alcohol use in this sample was low.
Tyrlík and Konecný (2011) Czech Republic	3388 mothers	Cross-sectional	18 months	Moderate alcohol consumption (regularity and frequency).	Mothers’ activities with children and mother–child relationship ^GP^	Maternal moderate drinking was associated with impaired parenting behaviors. Abstinent mothers expressed more concern and emotions for the child and gave more attention to their child’s needs and educational activities. There were no significant differences in the frequency of physical activities with the child (cuddling, physical playing, and walks).
Van der Vorst et al. (2006) The Netherlands	416 families	Longitudinal, 3 waves (annual)	13 to 16 years (two siblings)	Parental alcohol consumption (frequency and intensity).	Alcohol-specific rules ^AP^	Parental drinking was related to fewer rules for both adolescents, but only the associations between the alcohol consumption of fathers and having alcohol-specific rules were significant.
Wolf and Freisthler (2025) USA	234 mothers	Longitudinal 3 waves over consecutive years (2020 to 2022)	2 to 12 years	Maternal alcohol use: Drinking frequency; average drinks per occasion; total volume in a period.	Aggressive discipline: Psychological aggression; corporal punishment.	Maternal alcohol consumption was a significant predictor of aggressive discipline. Specifically, higher drinking frequency was significantly associated with increased odds of weekly aggressive parenting behaviors (OR = 1.049). Total volume was only driven by frequency of drinking. These values decreased after pandemic time but statistical link between frequent alcohol use and aggressive discipline remained over the two-year period.
Wolf et al. (2021) USA	329 parents	Cross-sectional	2 to 12 years	Parental alcohol use frequency.	Discipline: punitive parenting ^GP^	The frequency of alcohol use was initially associated with punitive parenting; however, this association was not significant with demographic covariates. Parents who drank alcohol both monthly and weekly and had higher stress were more likely to carry out punitive parenting than non-drinking parents with high stress.
Zhang et al. (1999) USA	378 adolescents (male)	Cross-sectional	16 to 19 years	Parental alcohol consumption.	Parental closeness ^GP^	Mothers’ drinking had no significant effect on closeness to the parent, whereas fathers’ drinking significantly and negatively affected paternal closeness (β = −0.10, *p* < 0.05).

AUD: Alcohol use disorder; ^GP^ General parenting; ^AP^ Alcohol-specific parenting; Families—one mother, father and child (exceptions are indicated).

## Data Availability

The original contributions presented in this study are included in the article/Supplementary Material. Further inquiries can be directed to the corresponding author.

## Supplementary Materials

The following supporting information can be downloaded at: https://www.mdpi.com/article/10.3390/bs16020236/s1, Table S1: Prisma 2020 for Abstracts Checklist, Table S2: Prisma 2020 Checklist, Table S3: Risk of Bias Assessment, Table S4: Studies Excluded on Critical Appraisal. List S1: References of Included Studies.

## Abbreviations

The following abbreviations are used in this manuscript:

AP	Alcohol-specific parenting
AUD	Alcohol Use Disorder
CASP	Critical Appraisal Skills Programme
GP	General parenting
NDA	Non-dependent alcohol use;
PRISMA	Preferred Reporting Items for Systematic Reviews and Meta-Analyses
WEIRD	Western, Educated, Industrialized, Rich, and Democratic

## Appendix A. Description of Search Terms

Full search terms for databases searched through Ovid: Embase, MEDLINE, and PsycINFO. The database search was developed in consultation with a librarian at the University of Agder in Norway.

1
((drink or drinking or alcohol* or wine or beer) adj7 (parent* or mother* or father* or guardian* or custodian* or maternal* or paternal*)).ti,ab.

2
parenting.ti,ab,hw.

3
((parent* or mother* or father* or maternal* or family or families) adj4 (bond* or practic* or factor* or model* or style* or dimension* or monitor* or behavio* or involvement* or routin* or rule* or warm* or relation* or support* or emotion* or role* or communicat* or characteristic* or attitude* or permiss* or approv* or suppl* or provision* or availabil* or control* or disciplin* or neglect* or abuse* or control* or conflict* or punish* or affection* or guidance* or hostil* or closeness* or attach* or violen* or interact* or ritual* or function*)).ti,ab.

1 AND (2 OR 3)

## Appendix B. Definitions of Parenting Factors

**Parenting Factors**	**Definition**
**Parental alcohol use**	
	The frequency and/or intensity of parents’ alcohol-drinking behaviors. These behaviors can be observed and learned by their child or occur without the child being present. Parental alcohol use/consumption/drinking includes, but is not limited to, different variables of problematic drinking, such as intoxication, binge drinking, heavy episodic drinking, dependency, and other alcohol-related problems. No lower alcohol use threshold is applied.
**Alcohol-specific parenting practices**	
Alcohol-specific communication	Parent–child discussions about alcohol that can cover numerous topics, such as the adverse effects/dangers of drinking, alcohol in the media, and personal experiences of alcohol.
Parental attitudes toward alcohol use	The child’s perception of their parents’ attitudes toward alcohol and to whether parents approve or disapprove of their own adolescent drinking behavior.
Provision of alcohol	If parents make alcohol available or allow their child to drink alcohol at home.
Rules about alcohol	The rules established by the parents prohibiting alcohol use, preventing unsupervised drinking, and/or limiting their child’s drinking.
**General parenting practices**	
Family conflict/abuse	The amount of hostility, conflict, criticism, and relational tension within the family. All forms of abuse, violence, and neglect are included.
General communication	The frequency, quality, and/or satisfaction of the communication between the child/adolescent and their parents. Conversations can concern either general information or highly emotional topics.
Parental discipline	Actions by parents to regulate and direct their child’s behavior. It can include setting rules, establishing consequences, and applying those consequences when rules are broken. Discipline can differ in the consistency, amount, and strictness of the established rules.
Parental involvement	The frequency with which parents and children are involved in shared activities such as hobbies, tasks, watching television, playing games, and having dinner together.
Parental monitoring	Parents’ knowledge of their child’s activities, whereabouts, and friends.
Parental support	Extent to which the child feels their parents provide emotional and instrumental support such as love, encouragement, acceptance, praise, help, and guidance.
Parent–child relationship quality	The level of warmth, mutual attachment, bonding, affection, and other positive interactions perceived by the child and parents.
Factors and definitions based on the review by Ryan et al. (2010) and revised by Yap et al. (2017)	
