# Supplementary material for: Associations Between Parental Alcohol Use and Parenting Practices: A Systematic Review
Source: Behav Sci (Basel). 2026 Feb 7;16(2):236. doi: 10.3390/bs16020236 (PMC12938130; doi:10.3390/bs16020236)
Supplement: Supplementary file 1 [file behavsci-16-00236-s001.zip › behavsci-3950311-supplementary.pdf]

Supplementary Material

Associations between parental alcohol use and parenting practices: a systematic review

Contents:

Table S1: Prisma 2020 for Abstract Checklist ………………………………………………………………… **1**

Table S2: Prisma 2020 Checklist ……………………………………………………………………………….. **2**

List S1: References of Included Studies ……………………………………………………………………... **5**

Table S3: Risk of Bias Assessment ………………………………………………………………………….. **9**

Table S4: Studies Excluded on Critical Appraisal ……………………………………………………………….. **17**

Table S1: Prisma 2020 for Abstracts Checklist

Section and Topic	Item #	Checklist item	Reported (Yes/No)*
**TITLE**			
Title	1	Identify the report as a systematic review.	Yes
**BACKGROUND**			
Objectives	2	Provide an explicit statement of the main objective(s) or question(s) the review addresses.	Yes
**METHODS**			
Eligibility criteria	3	Specify the inclusion and exclusion criteria for the review.	Yes
Information sources	4	Specify the information sources (e.g., databases, registers) used to identify studies and the date when each was last searched.	Yes
Risk of bias	5	Specify the methods used to assess risk of bias in the included studies.	Yes
Synthesis of results	6	Specify the methods used to present and synthesise results.	Yes
**RESULTS**			
Included studies	7	Give the total number of included studies and participants and summarise relevant characteristics of studies.	Yes
Synthesis of results	8	Present results for main outcomes, preferably indicating the number of included studies and participants for each. If meta-analysis was done, report the summary estimate and confidence/credible interval. If comparing groups, indicate the direction of the effect (i.e. which group is favoured).	Yes
**DISCUSSION**			
Limitations of evidence	9	Provide a brief summary of the limitations of the evidence included in the review (e.g. study risk of bias, inconsistency and imprecision).	Yes
Interpretation	10	Provide a general interpretation of the results and important implications.	Yes
**OTHER**			
Funding	11	Specify the primary source of funding for the review.	Yes
Registration	12	Provide the register name and registration number.	Yes

* Some PRISMA items may be reported and described in a different location.

From: Page MJ, McKenzie JE, Bossuyt PM, Boutron I, Hoffmann TC, Mulrow CD, et al. The PRISMA 2020 statement: an updated guideline for reporting systematic reviews. BMJ 2021;372:n71. doi: 10.1136/bmj.n71

For more information, visit: http://www.prisma-statement.org/

Table S2: Prisma 2020 Checklist

Section and Topic	Item #	Checklist item	Location where item is reported
**TITLE**			
Title	1	Identify the report as a systematic review.	Page 1
**ABSTRACT**			
Abstract	2	See the PRISMA 2020 for Abstracts checklist.	Checklist included.
**INTRODUCTION**			
Rationale	3	Describe the rationale for the review in the context of existing knowledge.	Page 2
Objectives	4	Provide an explicit statement of the objective(s) or question(s) the review addresses.	Page 2
**METHODS**			
Eligibility criteria	5	Specify the inclusion and exclusion criteria for the review and how studies were grouped for the syntheses.	Page 3
Information sources	6	Specify all databases, registers, websites, organisations, reference lists and other sources searched or consulted to identify studies. Specify the date when each source was last searched or consulted.	Page 3
Search strategy	7	Present the full search strategies for all databases, registers and websites, including any filters and limits used.	Appendix A
Selection process	8	Specify the methods used to decide whether a study met the inclusion criteria of the review, including how many reviewers screened each record and each report retrieved, whether they worked independently, and if applicable, details of automation tools used in the process.	Page 3-4
Data collection process	9	Specify the methods used to collect data from reports, including how many reviewers collected data from each report, whether they worked independently, any processes for obtaining or confirming data from study investigators, and if applicable, details of automation tools used in the process.	Page 2-4
Data items	10a	List and define all outcomes for which data were sought. Specify whether all results that were compatible with each outcome domain in each study were sought (e.g., for all measures, time points, analyses), and if not, the methods used to decide which results to collect.	Table 1.; Appendix B.
	10b	List and define all other variables for which data were sought (e.g., participant and intervention characteristics, funding sources). Describe any assumptions made about any missing or unclear information.	Table 1.; Appendix B.
Study risk of bias assessment	11	Specify the methods used to assess risk of bias in the included studies, including details of the tool(s) used, how many reviewers assessed each study and whether they worked independently, and if applicable, details of automation tools used in the process.	Page 3-4; Sup. material.
Effect measures	12	Specify for each outcome the effect measure(s) (e.g., risk ratio, mean difference) used in the synthesis or presentation of results.	Table 1
Synthesis methods	13a	Describe the processes used to decide which studies were eligible for each synthesis (e.g., tabulating the study intervention characteristics and comparing against the planned groups for each synthesis (item #5)).	Page 4; Table 1.
	13b	Describe any methods required to prepare the data for presentation or synthesis, such as handling of missing summary statistics, or data conversions.	N/A
	13c	Describe any methods used to tabulate or visually display results of individual studies and syntheses.	Page 4; Table 1.
	13d	Describe any methods used to synthesize results and provide a rationale for the choice(s). If meta-analysis was performed, describe the model(s), method(s) to identify the presence and extent of statistical heterogeneity, and software package(s) used.	Page 4
	13e	Describe any methods used to explore possible causes of heterogeneity among study results (e.g., subgroup analysis, meta-regression).	N/A
	13f	Describe any sensitivity analyses conducted to assess robustness of the synthesized results.	N/A

Section and Topic	Item #	Checklist item	Location where item is reported
Reporting bias assessment	14	Describe any methods used to assess risk of bias due to missing results in a synthesis (arising from reporting biases).	N/A
Certainty assessment	15	Describe any methods used to assess certainty (or confidence) in the body of evidence for an outcome.	N/A
**RESULTS**			
Study selection	16a	Describe the results of the search and selection process, from the number of records identified in the search to the number of studies included in the review, ideally using a flow diagram.	Figure 1.
	16b	Cite studies that might appear to meet the inclusion criteria, but which were excluded, and explain why they were excluded.	Page 4; Sup. material; Figure 1;
Study characteristics	17	Cite each included study and present its characteristics.	Table 1. Sup. Material.
Risk of bias in studies	18	Present assessments of risk of bias for each included study.	Page 4; Sup. material.
Results of individual studies	19	For all outcomes, present, for each study: (a) summary statistics for each group (where appropriate) and (b) an effect estimate and its precision (e.g., confidence/credible interval), ideally using structured tables or plots.	Table 1.
Results of syntheses	20a	For each synthesis, briefly summarise the characteristics and risk of bias among contributing studies.	Pages 5-8; Table 1 Sup. material.
	20b	Present results of all statistical syntheses conducted. If meta-analysis was done, present for each the summary estimate and its precision (e.g. confidence/credible interval) and measures of statistical heterogeneity. If comparing groups, describe the direction of the effect.	N/A
	20c	Present results of all investigations of possible causes of heterogeneity among study results.	N/A
	20d	Present results of all sensitivity analyses conducted to assess the robustness of the synthesized results.	N/A
Reporting biases	21	Present assessments of risk of bias due to missing results (arising from reporting biases) for each synthesis assessed.	Sup. material.
Certainty of evidence	22	Present assessments of certainty (or confidence) in the body of evidence for each outcome assessed.	N/A
**DISCUSSION**			
Discussion	23a	Provide a general interpretation of the results in the context of other evidence.	Pages 5-8
	23b	Discuss any limitations of the evidence included in the review.	Pages 5-9
	23c	Discuss any limitations of the review processes used.	Pages 8-9
	23d	Discuss implications of the results for practice, policy, and future research.	Pages 8-9
**OTHER INFORMATION**			
Registration and protocol	24a	Provide registration information for the review, including register name and registration number, or state that the review was not registered.	3
	24b	Indicate where the review protocol can be accessed, or state that a protocol was not prepared.	3
	24c	Describe and explain any amendments to information provided at registration or in the protocol.	See protocol.
Support	25	Describe sources of financial or non-financial support for the review, and the role of the funders or sponsors in the review.	Page 4

Section and Topic	Item #	Checklist item	Location where item is reported
Competing interests	26	Declare any competing interests of review authors.	Page 9
Availability of data, code and other materials	27	Report which of the following are publicly available and where they can be found: template data collection forms; data extracted from included studies; data used for all analyses; analytic code; any other materials used in the review.	Page 9

From: Page MJ, McKenzie JE, Bossuyt PM, Boutron I, Hoffmann TC, Mulrow CD, et al. The PRISMA 2020 statement: an updated guideline for reporting systematic reviews. BMJ 2021;372:n71. doi: 10.1136/bmj.n71

For more information, visit: http://www.prisma-statement.org/

List S1: References of Included Studies

1 Anda RF, Whitfield CL, Felitti VJ et al. Adverse childhood experiences, alcoholic parents, and later risk of alcoholism and depression. *Psychiatr Serv* 2002; **53**: 1001–9. https://doi.org/10.1176/appi.ps.53.8.1001.

2 Au WM, Ho SY, Wang MP et al. Correlates of pro-drinking practices in drinking parents of adolescents in Hong Kong. *PLOS ONE* 2015; **10**: e0119554. https://doi.org/10.1371/journal.pone.0119554.

3 Au WM, Ho SY, Wang MP et al. Alcohol drinking and pro-drinking practices in parents of Hong Kong adolescents. *Alcohol Alcohol* 2014; **49**: 668–74. https://doi.org/10.1093/alcalc/agu063.

4 Bijttebier P, Goethals E. Parental drinking as a risk factor for children's maladjustment: The mediating role of family environment. *Psychol Addict Behav* 2006; **20**: 126–30. https://doi.org/10.1037/0893-164X.20.2.126.

5 Bryant L, MacKintosh AM, Bauld L. An exploration of the impact of non-dependent parental drinking on children. *Alcohol Alcohol* 2020; **55**: 121–7. https://doi.org/10.1093/alcalc/agz086.

6 Chassin L, Curran PJ, Hussong AM, Colder CR. The relation of parent alcoholism to adolescent substance use: A longitudinal follow-up study. *J Abnorm Psychol* 1996; **105**: 70–80. https://doi.org/10.1037//0021-843x.105.1.70.

7 Chassin L, Pillow DR, Curran PJ, Molina BSG, Barrera M, Jr. Relation of parental alcoholism to early adolescent substance use: A test of three mediating mechanisms. *J Abnorm Psychol* 1993; **102**: 3–19. https://doi.org/10.1037//0021-843x.102.1.3.

8 Edwards EP, Eiden RD, Leonard KE. Impact of fathers' alcoholism and associated risk factors on parent–infant attachment stability from 12 to 18 months. *Infant Ment Health J* 2004; **25**: 556–79. https://doi.org/10.1002/imhj.20027.

9 Edwards EP, Homish GG, Eiden RD, Grohman KK, Leonard KE. Longitudinal prediction of early childhood discipline styles among heavy drinking parents. *Addict Behav* 2009; **34**: 100–6. https://doi.org/10.1016/j.addbeh.2008.08.006.

10 Eiden RD, Chavez F, Leonard KE. Parent–infant interactions among families with alcoholic fathers. *Dev Psychopathol* 1999; **11**: 745–62. https://doi.org/10.1017/S0954579499002308.

11 Eiden RD, Colder C, Edwards EP, Leonard KE. A longitudinal study of social competence among children of alcoholic and nonalcoholic parents: Role of parental psychopathology, parental warmth, and self-regulation. *Psychol Addict Behav* 2009; **23**: 36–46. https://doi.org/10.1037/a0014839.

12 Eiden RD, Edwards EP, Leonard KE. Predictors of effortful control among children of alcoholic and nonalcoholic fathers. *J Stud Alcohol* 2004; **65**: 309–19. https://doi.org/10.15288/jsa.2004.65.309.

13 Eiden RD, Edwards EP, Leonard KE. Mother–infant and father–infant attachment among alcoholic families. *Dev Psychopathol* 2002; **14**: 253–78. https://doi.org/10.1017/S0954579402002043.

14 Eiden RD, Edwards EP, Leonard KE. A conceptual model for the development of externalizing behavior problems among kindergarten children of alcoholic families: Role of parenting and children's self-regulation. *Dev Psychol* 2007; **43**: 1187–201. https://doi.org/10.1037/0012-1649.43.5.1187.

15 Eiden RD, Leonard KE. Paternal alcoholism, parental psychopathology, and aggravation with infants. *J Subst Abuse* 2000; **11**: 17–29. https://doi.org/10.1016/S0899-3289(99)00016-4.

16 Eiden RD, Leonard KE, Hoyle RH, Chavez F. A transactional model of parent–infant interactions in alcoholic families. *Psychol Addict Behav* 2004; **18**: 350–61. https://doi.org/10.1037/0893-164X.18.4.350.

17 Eiden RD, Molnar DS, Colder C, Edwards EP, Leonard KE. A conceptual model predicting internalizing problems in middle childhood among children of alcoholic and nonalcoholic fathers: The role of marital aggression. *J Stud Alcohol Drugs* 2009; **70**: 741–50. https://doi.org/10.15288/jsad.2009.70.741.

18 Elam KK, Sternberg A, Waddell JT, Blake AJ, Chassin L. Mother and father prescription opioid misuse, alcohol use disorder, and parent knowledge in pathways to adolescent alcohol use. *J Youth Adolesc* 2020; **49**: 1663–73. https://doi.org/10.1007/s10964-020-01266-2.

19 Famularo R, Kinscherff R, Fenton T. Parental substance abuse and the nature of child maltreatment. *Child Abuse Negl* 1992; **16**: 475–83. https://doi.org/10.1016/0145-2134(92)90064-X.

20 Finan LJ, Schulz J, Gordon MS, Ohannessian CM. Parental problem drinking and adolescent externalizing behaviors: The mediating role of family functioning. *J Adolesc* 2015; 43: 100–10. https://doi.org/10.1016/j.adolescence.2015.05.001.

21 Finger B, Kachadourian LK, Molnar DS, Eiden RD, Edwards EP, Leonard KE. Alcoholism, associated risk factors, and harsh parenting among fathers: Examining the role of marital aggression. *Addict Behav* 2010; **35**: 541–8. https://doi.org/10.1016/j.addbeh.2009.12.029.

22 Freisthler B, Wolf JP. Testing a social mechanism: Does alcohol outlet density moderate the relationship between levels of alcohol use and child physical abuse? *Violence Vict* 2016; **31**: 1080–99. https://doi.org/10.1891/0886-6708.VV-D-14-00183.
23 Freisthler B, Wolf JP, Johnson-Motoyama M. Understanding the role of context-specific drinking in neglectful parenting behaviors. *Alcohol Alcohol* 2015; **50**: 542–50. https://doi.org/10.1093/alcalc/agv031.
24 Freisthler B, Johnson-Motoyama M, Kepple NJ. Inadequate child supervision: The role of alcohol outlet density, parent drinking behaviors, and social support. *Child Youth Serv Rev* 2014; 43: 75–84. https://doi.org/10.1016/j.childyouth.2014.05.002.
25 Freisthler B, Wolf JP, Hodge AI, Cao Y. Alcohol use and harm to children by parents and other adults. *Child Maltreat* 2020; **25**: 277–88. https://doi.org/10.1177/1077559519878514.
26 Handley ED, Chassin L. Alcohol-specific parenting as a mechanism of parental drinking and alcohol use disorder risk on adolescent alcohol use onset. *J Stud Alcohol Drugs* 2013; **74**: 684–93. https://doi.org/10.15288/jsad.2013.74.684.
27 Jacob T, Krahn GL, Leonard K. Parent–child interactions in families with alcoholic fathers. *J Consult Clin Psychol* 1991; **59**: 176–81; discussion 183. https://doi.org/10.1037/0022-006X.59.1.176.
28 Jacob T, Leonard KE, Randolph Haber JR. Family interactions of alcoholics as related to alcoholism type and drinking condition. *Alcohol Clin Exp Res* 2001; **25**: 835–43. https://doi.org/10.1111/j.1530-0277.2001.tb02287.x.
29 Jacques DT, Sturge-Apple ML, Davies PT, Cicchetti D. Parsing alcohol-dependent mothers' insensitivity to child distress: Longitudinal links with children's affective and anxiety problems. *Dev Psychol* 2021; **57**: 900–12. https://doi.org/10.1037/dev0001190.
30 Jacques DT, Sturge-Apple ML, Davies PT, Cicchetti D. Maternal alcohol dependence and harsh caregiving across parenting contexts: The moderating role of child negative emotionality. *Dev Psychopathol* 2020; **32**: 1509–23. https://doi.org/10.1017/S0954579419001445.
31 Kachadourian LK, Eiden RD, Leonard KE. Paternal alcoholism, negative parenting, and the mediating role of marital satisfaction. *Addict Behav* 2009; **34**: 918–27. https://doi.org/10.1016/j.addbeh.2009.05.003.
32 Keller PS, Michlitsch H, Rawn KP. Retrospective reports of parental problem drinking and parent reactions to child negative emotions: Implications for emotion regulation in the transition to adulthood. *Emerg Adult* 2021; **10**: 620-31. https://doi.org/10.1177/21676968211024401.
33 Kelley ML, Pearson MR, Trinh S, Klostermann K, Krakowski K. Maternal and paternal alcoholism and depressive mood in college students: Parental relationships as mediators of ACOA-depressive mood link. *Addict Behav* 2011; **36**: 700–6. https://doi.org/10.1016/j.addbeh.2011.01.028.
34 Kim HK, Pears KC, Fisher PA, Connelly CD, Landsverk JA. Trajectories of maternal harsh parenting in the first 3 years of life. *Child Abuse Negl* 2010; **34**: 897–906. https://doi.org/10.1016/j.chiabu.2010.06.002.
35 Lang AR, Pelham WE, Atkeson BM, Murphy DA. Effects of alcohol intoxication on parenting behavior in interactions with child confederates exhibiting normal or deviant behaviors. *J Abnorm Child Psychol* 1999; **27**: 177–89. https://doi.org/10.1023/a:1021996122095.
36 Lee SJ, Perron BE, Taylor CA, Guterman NB. Paternal psychosocial characteristics and corporal punishment of their 3-year-old children. *J Interpers Violence* 2011; **26**: 71–87. https://doi.org/10.1177/0886260510362888.
37 Lloyd MH, Kepple NJ. Unpacking the parallel effects of parental alcohol misuse and low income on risk of supervisory neglect. *Child Abuse Negl* 2017; 69: 72–84. https://doi.org/10.1016/j.chiabu.2017.03.007.
38 Maggs JL, Staff JA. Parents who allow early adolescents to drink *J Adolesc Health.* 2018; **62**: 245–7. https://doi.org/10.1016/j.jadohealth.2017.09.016.
39 Maggs JL, Cassinat JR, Kelly BC, Mustillo SA, Whiteman SD. Parents who first allowed adolescents to drink alcohol in a family context during spring 2020 COVID-19 emergency shutdowns. *J Adolesc Health* 2021; **68**: 816–8. https://doi.org/10.1016/j.jadohealth.2021.01.010.
40 Mares SHW, Van der Vorst H, Engels RCME, Lichtwarck-Aschoff A. Parental alcohol use, alcohol-related problems, and alcohol-specific attitudes, alcohol-specific communication, and adolescent excessive alcohol use and alcohol-related problems: An indirect path model. *Addict Behav* 2011; **36**: 209–16. https://doi.org/10.1016/j.addbeh.2010.10.013.
41 Moser RP, Jacob T. Parent–child interactions and child outcomes as related to gender of alcoholic parent. *J Subst Abuse* 1997; 9: 189–208. https://doi.org/10.1016/S0899-3289(97)90016-X.
42 Ohannessian CM. Parental problem drinking and adolescent psychosocial adjustment: The mediating role of adolescent-parent communication. *J Res Adolesc* 2012; **22**: 498–511. https://doi.org/10.1111/j.1532-7795.2012.00791.x.
43 Rangarajan S. Mediators and moderators of parental alcoholism effects on offspring self-esteem. *Alcohol Alcoholism* 2008; **43**: 481–91. https://doi.org/10.1093/alcalc/agn034.

44 Roberts R, Beckwith M, Watts D. Mothers' intentions to introduce their adolescent to alcohol use: Does mothers' alcohol use effect intentions? *Aust N Z J Public Health* 2010; **34**: 281–7. https://doi.org/10.1111/j.1753-6405.2010.00527.x.
45 Rochat TJ, Houle B, Stein A, Mitchell J, Bland RM. Maternal alcohol use and children's emotional and cognitive outcomes in rural South Africa. *S Afr Med J* 2019; **109**: 526–34. https://doi.org/10.7196/SAMJ.2019.v109i7.13120.
46 Rutherford MJ, Cacciola JS, Alterman AI, McKay JR, Cook TJ. Young men's perceived quality of parenting based on familial history of alcoholism. *J Child Adolesc Subst Abuse* 1998; **6**: 43–56. https://doi.org/10.1300/J029v06n03_03.
47 Schacht PM, Cummings EM, Davies PT. Fathering in family context and child adjustment: A longitudinal analysis. *J Fam Psychol* 2009; **23**: 790–7. https://doi.org/10.1037/a0016741.
48 Senchak M, Leonard KE, Greene BW, Carroll A. Comparisons of adult children of alcoholic, divorced, and control parents in four outcome domains. *Psychol Addict Behav* 1995; **9**: 147–56. https://doi.org/10.1037/0893-164X.9.3.147.
49 Smyth BP, Darker CD, Donnelly-Swift E, Barry JM, Allwright SP. A telephone survey of parental attitudes and behaviours regarding teenage drinking. *BMC Public Health* 2010; **10**: 297. https://doi.org/10.1186/1471-2458-10-297.
50 Spieker SJ, Gillmore MR, Lewis SM, Morrison DM, Lohr MJ. Psychological distress and substance use by adolescent mothers: Associations with parenting attitudes and the quality of mother–child interaction. *J Psychoact Drugs* 2001; **33**: 83–93. https://doi.org/10.1080/02791072.2001.10400472.
51 Su J, Kuo SI, Aliev F, et al. Influence of parental alcohol dependence symptoms and parenting on adolescent risky drinking and conduct problems: A family systems perspective. *Alcohol Clin Exp Res* 2018; **42**: 1783–94. https://doi.org/10.1111/acer.13827.
52 Taber-Thomas SM, Knutson JF. Association between mothers' alcohol use histories and deficient parenting in an economically disadvantaged sample. *Child Maltreat* 2021; **26**: 40–9. https://doi.org/10.1177/1077559520925550.
53 Tweed SH, Ryff CD. Family climate and parent–child relationships: Recollections from a nonclinical sample of adult children of alcoholic fathers. *Res Nurs Health* 1996; **19**: 311–21. https://doi.org/10.1002/(SICI)1098-240X(199608)19:4<311::AID-NUR5>3.0.CO;2-L.
54 Tyrlík M, Konecný S. Moderate alcohol consumption as a mediator of mother's behaviour towards her child. *Cent Eur J Public Health* 2011; **19**: 143–6. https://doi.org/10.21101/cejph.a3665.
55 Van der Vorst H, Engels RC, Meeus W, Deković M. The impact of alcohol-specific rules, parental norms about early drinking and parental alcohol use on adolescents' drinking behavior. *J Child Psychol Psychiatry* 2006; **47**: 1299–306. https://doi.org/10.1111/j.1469-7610.2006.01680.x.
56 Van der Zwaluw CS, Scholte RH, Vermulst AA, Buitelaar JK, Verkes RJ, Engels RC. Parental problem drinking, parenting, and adolescent alcohol use. *J Behav Med* 2008; **31**: 189–200. https://doi.org/10.1007/s10865-007-9146-z.
57 Ward BM, Snow PC. Factors affecting parental supply of alcohol to underage adolescents. *Drug Alcohol Rev* 2011; **30**: 338–43. https://doi.org/10.1111/j.1465-3362.2010.00228.x.
58 Wolf JP, Freisthler B, Chadwick CC. Stress, alcohol use, and punitive parenting during the COVID-19 pandemic. *Child Abuse Negl* 2021; **117**: 105090. https://doi.org/10.1016/j.chiabu.2021.105090.
59 Zhang L, Welte JW, Wieczorek WF. The influence of parental drinking and closeness on adolescent drinking. *J Stud Alcohol* 1999; **60**: 245–51. https://doi.org/10.15288/jsa.1999.60.245.
60 Amundsen, V. R., Gjesmoe, A., Andreassen, E., Bølstad, E., Severinsen, Y., Viana, K., Bjørk, R. F., Nygaard, E., Bekkhus, M., & Tsotsi, S. (2025). Alcohol use and parental socialization of emotion in a population-based sample. *Parenting*, *25*(1). https://doi.org/10.1080/15295192.2025.2465988
61 Freisthler, B., Sarabia, J., & Price Wolf, J. (2023). Using ecological momentary assessments to understand how drinking during special occasions relates to parenting behaviors. *Alcohol: Clinical and Experimental Research*, *47*(12), 2343–2353. https://doi.org/10.1111/acer.15206
62 Freisthler, B., & Wolf, J. (2023). Sequencing drinking events and use of punitive, nonpunitive, and positive parenting behaviors with ecological momentary assessment. *Drug and Alcohol Dependence*, *242*, Article 109716. https://doi.org/10.1016/j.drugalcdep.2022.109716
63 Jacques, D. T., Sturge-Apple, M. L., Davies, P. T., & Cicchetti, D. (2025). Maternal alcohol dependence symptoms, maternal insensitivity to children's distress, and young children's blunted emotional reactivity. *Development and Psychopathology*, *37*, 555–577. https://doi.org/10.1017/S0954579424000324

64 Keller, P. S., Rawn, K. P., Dunsmore, J., & Zvolensky, M. (2024). Parental problem drinking, parent emotion socialization and child emotion regulation. *Journal of Applied Developmental Psychology*, *95*, Article 101724. https://doi.org/10.1016/j.appdev.2024.101724

65 Keller, P. S., Rawn, K. P., Dunsmore, J. C., Zvolensky, M., & El-Sheikh, M. (2023). Parental drinking and observations of parent–child problem-solving discussions: Do drinking motives matter? *Journal of Family Psychology*, *37*(7), 993–1004. https://doi.org/10.1037/fam0001135

66 Laslett, A.-M., Kuntsche, S., Wilson, I. M., Taft, A., Fulu, E., Jewkes, R., & Graham, K. (2022). The relationship between fathers' heavy episodic drinking and fathering involvement in five Asia-Pacific countries: An individual participant data meta-analysis. *Alcoholism: Clinical and Experimental Research*, *46*(12), 2137–2148. https://doi.org/10.1111/acer.14955

67 Seay, K. D. (2026). Pathways from parent substance misuse to child and adolescent trauma. *Children and Youth Services Review*, *181*, Article 108724. https://doi.org/10.1016/j.childyouth.2025.108724

68 Wolf, J. P., & Freisthler, B. (2025). Maternal drinking, stress and use of aggressive parenting over the course of the COVID-19 pandemic. *Alcohol and Alcoholism*, *60*(3), agaf020. https://doi.org/10.1093/alcalc/agaf020

Table S3: Risk of Bias Assessment

Questions from the Critical Appraisal Skills Programme (CASP)[1,2]
Cohort study and case control study checklists

1. Did the study address a clearly focused issue?
2. Were the cases or the cohort recruited in an acceptable way?
3. Did the authors use an appropriate method to answer their question?
4. Were the controls selected in an acceptable way?
5. Was the exposure accurately measured to minimize bias?
6. Was the outcome accurately measured to minimize bias?
7. Have the authors identified all important confounding factors? Aside from the experimental intervention, were the groups treated equally?
8. Have the authors taken account of the potential confounding factors in the design and/or in their analysis?
9. Do you believe the results?
10. Do the results of this study fit with other available evidence?

The assessment was conducted independently by two reviewers (BC and SH) using guidance from the Critical Appraisal Skills Programme (CASP). A template was created based on modified CASP checklists and questions suitable for the study design. These were the response alternative to each question:

a) Yes
b) Unclear / Can't tell
c) No
d) Not applicable

There were prompts below the questions that highlight the issues and important guidelines to consider when answering.

Following the independent assessment, each study appraisal was compared and discussed together. It is worth noting that the reviewers considered the overall quality and relevance of the studies when making decisions regarding the critical appraisal. Since a strict cut-off for what constitutes a high risk of bias is not indicated, it was agreed and decided by the research team, that two or more "No" answers would rate this measure. Studies with a global final assessment of high risk of bias were not included in the narrative synthesis in this systematic review.

Table of risk of bias assessment

	Critical Appraisal Questions										
	1	2	3	4	5	6	7	8	9	10	Risk of bias
Amundsen et al. (2025) [71]	Yes	NA	Yes	NA	Yes	Yes	Yes	Yes	Yes	Yes	Low
Anda et al. (2002)[3]	Yes	NA	Yes	NA	Yes	Yes	Yes	Yes	Yes	Yes	Low
Au et al. (2015)[4]	Yes	NA	Yes	NA	Yes	Yes	Yes	Yes	Yes	Yes	Low
Au et al. (2014)[5]	Yes	NA	Yes	NA	Yes	Yes	Yes	Yes	Yes	Yes	Low
Bijttebier & Goethals (2006)[6]	Yes	NA	Yes	NA	Yes	Yes	No	Un	Yes	Yes	Moderate
Bryant et al. (2020)[7]	Yes	NA	Yes	NA	Un	Yes	Yes	Yes	Yes	Yes	Low
Chassin et al. (1996)[8]	Yes	Yes	Yes	Yes	Yes	NA	Un	Un	Yes	Yes	Moderate
Chassin et al. (1993)[9]	Yes	Yes	Yes	Yes	Yes	NA	Yes	Yes	Yes	Yes	Low
Edwards et al. (2004)[10]	Yes	Yes	Yes	Yes	Yes	NA	Yes	Yes	Un	Yes	Low
Edwards et al. (2009)[11]	Yes	Yes	Yes	Yes	Yes	NA	Yes	Yes	Yes	Yes	Low
Eiden et al. (1999)[12]	Yes	Yes	Yes	Yes	Yes	Yes	Yes	Yes	Un	Yes	Low
Eiden et al. (2009)[13]	Yes	Yes	Yes	Yes	Yes	Yes	Yes	Yes	Yes	Yes	Low
Eiden et al. (2004)[14]	Yes	Yes	Yes	Yes	Yes	Yes	Yes	Yes	Yes	Yes	Low
Eiden et al. (2002)[15]	Yes	Yes	Yes	NA	Yes	Yes	Yes	Yes	Yes	Yes	Low
Eiden et al. (2007)[16]	Yes	Yes	Yes	Yes	Yes	Yes	Yes	Yes	Yes	Yes	Low
Eiden et al. (2000)[17]	Yes	Yes	Yes	Yes	Yes	Yes	Yes	Yes	Yes	Yes	Low
Eiden et al. (2004)[18]	Yes	Yes	Yes	Yes	Yes	Yes	Yes	Yes	Yes	Yes	Low
Eiden et al. (2009)[19]	Yes	Yes	Yes	Yes	Yes	Yes	Yes	Yes	Yes	Yes	Low
Elam et al. (2020)[20]	Yes	Yes	Yes	Yes	Yes	Yes	Yes	Yes	Yes	Yes	Low
Famularo et al. (1992)[21]	Yes	Yes	Yes	Yes	Un	Yes	No	Un	Yes	Yes	Moderate
Finan et al.(2015)[22]	Yes	NA	Yes	NA	Yes	Yes	No	Un	Yes	Yes	Moderate
Finger et al. (2010)[23]	Yes	Yes	Yes	Yes	Yes	Yes	Yes	Yes	Yes	Yes	Low
Freisthler, Sarabia & Wolf (2023) [72]	Yes	Un	Yes	Yes	NA	Yes	Yes	Yes	Yes	Yes	Low
Freisthler & Wolf (2023) [73]	Yes	Yes	Yes	Yes	NA	Yes	Yes	Yes	Yes	Yes	Low
Freisthler & Wolf (2016)[24]	Yes	NA	Yes	NA	Yes	Yes	Yes	Yes	Yes	Yes	Low
Freisthler et al. (2015)[25]	Yes	NA	Yes	NA	Yes	Yes	Yes	Yes	Yes	Yes	Low
Freisthler et al. (2014)[26]	Yes	NA	Yes	NA	Yes	Yes	Yes	Yes	Yes	Yes	Low
Freisthler et al. (2020)[27]	Yes	NA	Un	NA	Yes	Yes	Yes	Yes	Un	No	Moderate
Handley & Chassin (2013)[28]	Yes	Yes	Yes	Yes	Yes	Yes	Yes	Yes	Yes	Yes	Low
Jacob et al. (1991)[29]	Yes	NA	Un	NA	Yes	Yes	Yes	Yes	Un	Un	Moderate
Jacob et al. (2001)[30]	Yes	NA	Un	NA	Yes	Yes	No	Un	Un	Un	Moderate

Jacques et al. (2025) [74]	Yes	Yes	Yes	NA	Yes	Yes	Yes	Yes	Yes	Yes	Low
Jacques et al. (2021)[31]	Yes	NA	Yes	NA	Yes	Yes	Yes	Yes	Yes	Yes	Low
Jacques et al. (2020)[32]	Yes	NA	Yes	NA	Yes	Yes	Yes	Yes	Yes	Yes	Low
Kachadourian et al. (2009)[33]	Yes	NA	Yes	NA	Yes	Yes	Yes	Yes	Yes	Yes	Low
Keller et al. (2024)[75]	Yes	Yes	Yes	NA	Yes	Yes	Yes	Yes	Yes	Yes	Low
Keller et al. (2023)[76]	Yes	Yes	Yes	NA	Yes	Yes	Yes	Yes	Yes	Yes	Low
Keller et al. (2021)[34]	Yes	NA	No	NA	Yes	Yes	Yes	Yes	Yes	Yes	Moderate
Kelley et al. (2011)[35]	Yes	Yes	NA	Yes	Yes	Yes	Un	No	Yes	Yes	Moderate
Kim et al. (2010)[36]	Yes	NA	Yes	NA	Yes	Yes	Yes	Yes	Yes	Yes	Low
Lang et al. (1999)[37]	Yes	Yes	Un	Un	Yes	Yes	Yes	Yes	Yes	Yes	Moderate
Laslett et al. (2022)[77]	Yes	Yes	Yes	Yes	Yes	Yes	Yes	Yes	Yes	Yes	Low
Lee et al. (2011)[38]	Yes	NA	Yes	NA	Yes	Yes	Yes	Yes	Yes	Yes	Low
Lloyd & Kepple (2017)[39]	Yes	NA	Yes	NA	Yes	Yes	Yes	Yes	Yes	Yes	Low
Maggs & Staff (2018)[40]	Yes	NA	Yes	NA	Yes	Yes	Yes	Yes	Yes	Yes	Low
Maggs et al. (2021)[41]	Yes	NA	Yes	NA	Un	Yes	Yes	Yes	Yes	Yes	Low
Mares et al. (2011)[42]	Yes	NA	Yes	NA	Yes	Yes	Un	No	Yes	Yes	Moderate
Moser & Jacob (1997)[43]	Yes	Yes	Yes	Yes	Yes	Yes	Yes	Un	Yes	Yes	Low
Ohannessian (2012)[44]	Yes	NA	Yes	NA	Yes	Yes	Yes	Yes	Yes	Yes	Low
Rangarajan (2008)[45]	Yes	NA	Yes	NA	Yes	Yes	Yes	Yes	Yes	Yes	Low
Roberts et al. (2010)[46]	Yes	NA	Yes	NA	Yes	Yes	No	Un	Yes	Un	Moderate
Rochat et al. (2019)[47]	Yes	NA	Yes	NA	Yes	Yes	Yes	No	Yes	Yes	Moderate
Rutherford et al. (1998)[48]	Yes	NA	Yes	NA	Yes	Yes	Yes	No	Un	Yes	Moderate
Schacht et al. (2009)[49]	Yes	NA	Yes	NA	Yes	Yes	Un	No	Yes	Yes	Moderate
Seay (2026)[78]	Yes	Yes	Yes	NA	Yes	Yes	Yes	Yes	Un	Un	Moderate
Senchak et al. (1995)[50]	Yes	Yes	Yes	Yes	Yes	NA	Yes	Yes	Yes	Yes	Low
Smyth et al. (2010)[51]	Yes	Un	Yes	NA	No	Un	Yes	Yes	Yes	Yes	Moderate
Spieker et al. (2001)[52]	Yes	NA	Yes	NA	Yes	Yes	Un	No	Yes	Yes	Moderate
Su et al. (2018)[53]	Yes	NA	Yes	NA	Yes	Yes	Yes	Yes	Yes	Yes	Low
Taber-Thomas & Knutson (2020)[54]	Yes	NA	Yes	NA	Yes	Yes	Yes	Yes	Yes	Yes	Low
Tweed & Ryff (1996)[55]	Yes	Yes	Yes	Yes	Yes	NA	Yes	Yes	Un	No	Moderate
Tyrlík & Konecný (2011)[56]	Yes	NA	Yes	NA	Yes	Yes	No	Un	Yes	Yes	Moderate
Van der Vorst et al. (2006)[57]	Yes	NA	Yes	NA	Yes	Yes	No	Un	Yes	Yes	Moderate
Van der Zwaluw et al. (2008)[58]	Yes	NA	Yes	NA	Yes	Yes	No	Un	Yes	Un	Moderate
Ward & Snow (2011)[59]	Yes	NA	Yes	NA	Yes	Yes	Yes	No	Yes	Yes	Moderate

Wolf & Freisthler (2025)[79]	Yes	Yes	Yes	NA	Yes	Yes	Yes	Yes	Yes	Yes	Low
Wolf et al. (2021)[60]	Yes	NA	Un	NA	Yes	Yes	Yes	Yes	Yes	Un	Moderate
Zhang et al. (1999)[61]	Yes	NA	Yes	NA	Yes	Yes	Yes	Yes	Yes	Yes	Low
Dumka & Roosa (1995)[62]	Yes		Yes		Yes	Yes	No	No	Un	Un	High
Dumka & Roosa (1993)[63]	Yes		Yes		Yes	Yes	No	No	Un	Un	High
Hyphantis et al. (1991)[64]	No		No	*	*	*	*	*	*	*	High
Mahato et al. (2009)[65]	No	No	*	*	*	*	*	*	*	*	High
Reich et al. (1988)[66]	No	No	*	*	*	*	*	*	*	*	High
Sebre et al. (2004)[67]	Yes		Yes		Un	Yes	No	No	Un	Un	High
Seilhamer et al. (1993)[68]	Yes	No	Un		Yes	Yes	No	No	No	Un	High
Stout & Mintz (1996)[69]	No	Un	No	*	*	*	*	*	*	*	High
Zincir et al. (2010)[70]	No		Un		No	*	*	*	*	*	High

Risk of bias final rating:
Low (only YES ratings with one Unclear at the most)
Moderate or unclear risk (one NO or more than one Unclear)
High (two or more NO ratings) – highlighted in red

Un: Unclear
NA: Not applicable
* Not worth proceeding

References:

1 Critical Appraisal Skills Programme. CASP Case Control Study Checklist. 2018. https://casp-uk.net/casp-tools-checklists/ (accessed March 28, 2022)

2 Critical Appraisal Skills Programme. CASP Cohort Study Checklist. 2018. https://casp-uk.net/casp-tools-checklists/ (accessed March 28, 2022)

3 Anda RF, Whitfield CL, Felitti VJ, et al. Adverse childhood experiences, alcoholic parents, and later risk of alcoholism and depression. *Psychiatr Serv* 2002; **53**: 1001–9. https://doi.org/10.1176/appi.ps.53.8.1001.

4 Au WM, Ho SY, Wang MP et al. Correlates of pro-drinking practices in drinking parents of adolescents in Hong Kong. *PLOS ONE* 2015; **10**: e0119554. https://doi.org/10.1371/journal.pone.0119554.

5 Au WM, Ho SY, Wang MP et al. Alcohol drinking and pro-drinking practices in parents of Hong Kong adolescents. *Alcohol Alcohol* 2014; **49**: 668–74. https://doi.org/10.1093/alcalc/agu063.

6 Bijttebier P, Goethals E. Parental drinking as a risk factor for children's maladjustment: The mediating role of family environment. *Psychol Addict Behav* 2006; **20**: 126–30. https://doi.org/10.1037/0893-164X.20.2.126.

7 Bryant L, MacKintosh AM, Bauld L. An exploration of the impact of non-dependent parental drinking on children. *Alcohol Alcohol* 2020; **55**: 121–7. https://doi.org/10.1093/alcalc/agz086.

8 Chassin L, Curran PJ, Hussong AM, Colder CR. The relation of parent alcoholism to adolescent substance use: A longitudinal follow-up study. *J Abnorm Psychol* 1996; **105**: 70–80. https://doi.org/10.1037//0021-843x.105.1.70.

9 Chassin L, Pillow DR, Curran PJ, Molina BSG, Barrera M, Jr. Relation of parental alcoholism to early adolescent substance use: A test of three mediating mechanisms. *J Abnorm Psychol* 1993; **102**: 3–19. https://doi.org/10.1037//0021-843x.102.1.3.

10 Edwards EP, Eiden RD, Leonard KE. Impact of fathers' alcoholism and associated risk factors on parent–infant attachment stability from 12 to 18 months. *Infant Ment Health J* 2004; **25**: 556–79. https://doi.org/10.1002/imhj.20027.

11 Edwards EP, Homish GG, Eiden RD, Grohman KK, Leonard KE. Longitudinal prediction of early childhood discipline styles among heavy drinking parents. *Addict Behav* 2009; **34**: 100–6. https://doi.org/10.1016/j.addbeh.2008.08.006.

12 Eiden RD, Chavez F, Leonard KE. Parent–infant interactions among families with alcoholic fathers. *Dev Psychopathol* 1999; **11**: 745–62. https://doi.org/10.1017/S0954579499002308.
13 Eiden RD, Colder C, Edwards EP, Leonard KE. A longitudinal study of social competence among children of alcoholic and nonalcoholic parents: Role of parental psychopathology, parental warmth, and self-regulation. *Psychol Addict Behav* 2009; **23**: 36–46. https://doi.org/10.1037/a0014839.
14 Eiden RD, Edwards EP, Leonard KE. Predictors of effortful control among children of alcoholic and nonalcoholic fathers. *J Stud Alcohol* 2004; **65**: 309–19. https://doi.org/10.15288/jsa.2004.65.309.
15 Eiden RD, Edwards EP, Leonard KE. Mother–infant and father–infant attachment among alcoholic families. *Dev Psychopathol* 2002; **14**: 253–78. https://doi.org/10.1017/S0954579402002043.
16 Eiden RD, Edwards EP, Leonard KE. A conceptual model for the development of externalizing behavior problems among kindergarten children of alcoholic families: Role of parenting and children's self-regulation. *Dev Psychol* 2007; **43**: 1187–201. https://doi.org/10.1037/0012-1649.43.5.1187.
17 Eiden RD, Leonard KE. Paternal alcoholism, parental psychopathology, and aggravation with infants. *J Subst Abuse* 2000; **11**: 17–29. https://doi.org/10.1016/S0899-3289(99)00016-4.
18 Eiden RD, Leonard KE, Hoyle RH, Chavez F. A transactional model of parent–infant interactions in alcoholic families. *Psychol Addict Behav* 2004; **18**: 350–61. https://doi.org/10.1037/0893-164X.18.4.350.
19 Eiden RD, Molnar DS, Colder C, Edwards EP, Leonard KE. A conceptual model predicting internalizing problems in middle childhood among children of alcoholic and nonalcoholic fathers: The role of marital aggression. *J Stud Alcohol Drugs* 2009; **70**: 741–50. https://doi.org/10.15288/jsad.2009.70.741.
20 Elam KK, Sternberg A, Waddell JT, Blake AJ, Chassin L. Mother and father prescription opioid misuse, alcohol use disorder, and parent knowledge in pathways to adolescent alcohol use. *J Youth Adolesc* 2020; **49**: 1663–73. https://doi.org/10.1007/s10964-020-01266-2.
21 Famularo R, Kinscherff R, Fenton T. Parental substance abuse and the nature of child maltreatment. *Child Abuse Negl* 1992; **16**: 475–83. https://doi.org/10.1016/0145-2134(92)90064-X.
22 Finan LJ, Schulz J, Gordon MS, Ohannessian CM. Parental problem drinking and adolescent externalizing behaviors: The mediating role of family functioning. *J Adolesc* 2015; 43: 100–10. https://doi.org/10.1016/j.adolescence.2015.05.001.
23 Finger B, Kachadourian LK, Molnar DS, Eiden RD, Edwards EP, Leonard KE. Alcoholism, associated risk factors, and harsh parenting among fathers: Examining the role of marital aggression. *Addict Behav* 2010; **35**: 541–8. https://doi.org/10.1016/j.addbeh.2009.12.029.
24 Freisthler B, Wolf JP. Testing a social mechanism: Does alcohol outlet density moderate the relationship between levels of alcohol use and child physical abuse? *Violence Vict* 2016; **31**: 1080–99. https://doi.org/10.1891/0886-6708.VV-D-14-00183.
25 Freisthler B, Wolf JP, Johnson-Motoyama M. Understanding the role of context-specific drinking in neglectful parenting behaviors. *Alcohol Alcohol* 2015; **50**: 542–50. https://doi.org/10.1093/alcalc/agv031.
26 Freisthler B, Johnson-Motoyama M, Kepple NJ. Inadequate child supervision: The role of alcohol outlet density, parent drinking behaviors, and social support. *Child Youth Serv Rev* 2014; 43: 75–84. https://doi.org/10.1016/j.childyouth.2014.05.002.
27 Freisthler B, Wolf JP, Hodge AI, Cao Y. Alcohol use and harm to children by parents and other adults. *Child Maltreat* 2020; **25**: 277–88. https://doi.org/10.1177/1077559519878514.
28 Handley ED, Chassin L. Alcohol-specific parenting as a mechanism of parental drinking and alcohol use disorder risk on adolescent alcohol use onset. *J Stud Alcohol Drugs* 2013; **74**: 684–93. https://doi.org/10.15288/jsad.2013.74.684.
29 Jacob T, Krahn GL, Leonard K. Parent–child interactions in families with alcoholic fathers. *J Consult Clin Psychol* 1991; **59**: 176–81; discussion 183. https://doi.org/10.1037/0022-006X.59.1.176.
30 Jacob T, Leonard KE, Randolph Haber JR. Family interactions of alcoholics as related to alcoholism type and drinking condition. *Alcohol Clin Exp Res* 2001; **25**: 835–43. https://doi.org/10.1111/j.1530-0277.2001.tb02287.x.
31 Jacques DT, Sturge-Apple ML, Davies PT, Cicchetti D. Parsing alcohol-dependent mothers' insensitivity to child distress: Longitudinal links with children's affective and anxiety problems. *Dev Psychol* 2021; **57**: 900–12. https://doi.org/10.1037/dev0001190.
32 Jacques DT, Sturge-Apple ML, Davies PT, Cicchetti D. Maternal alcohol dependence and harsh caregiving across parenting contexts: The moderating role of child negative emotionality. *Dev Psychopathol* 2020; **32**: 1509–23. https://doi.org/10.1017/S0954579419001445.
33 Kachadourian LK, Eiden RD, Leonard KE. Paternal alcoholism, negative parenting, and the mediating role of marital satisfaction. *Addict Behav* 2009; **34**: 918–27. https://doi.org/10.1016/j.addbeh.2009.05.003.

34 Keller PS, Michlitsch H, Rawn KP. Retrospective reports of parental problem drinking and parent reactions to child negative emotions: Implications for emotion regulation in the transition to adulthood. *Emerg Adult* 2021; **10**: 620-31. https://doi.org/10.1177/21676968211024401.
35 Kelley ML, Pearson MR, Trinh S, Klostermann K, Krakowski K. Maternal and paternal alcoholism and depressive mood in college students: Parental relationships as mediators of ACOA-depressive mood link. *Addict Behav* 2011; **36**: 700–6. https://doi.org/10.1016/j.addbeh.2011.01.028.
36 Kim HK, Pears KC, Fisher PA, Connelly CD, Landsverk JA. Trajectories of maternal harsh parenting in the first 3 years of life. *Child Abuse Negl* 2010; **34**: 897–906. https://doi.org/10.1016/j.chiabu.2010.06.002.
37 Lang AR, Pelham WE, Atkeson BM, Murphy DA. Effects of alcohol intoxication on parenting behavior in interactions with child confederates exhibiting normal or deviant behaviors. *J Abnorm Child Psychol* 1999; **27**: 177–89. https://doi.org/10.1023/a:1021996122095.
38 Lee SJ, Perron BE, Taylor CA, Guterman NB. Paternal psychosocial characteristics and corporal punishment of their 3-year-old children. *J Interpers Violence* 2011; **26**: 71–87. https://doi.org/10.1177/0886260510362888.
39 Lloyd MH, Kepple NJ. Unpacking the parallel effects of parental alcohol misuse and low income on risk of supervisory neglect. *Child Abuse Negl* 2017; 69: 72–84. https://doi.org/10.1016/j.chiabu.2017.03.007.
40 Maggs JL, Staff JA. Parents who allow early adolescents to drink *J Adolesc Health.* 2018; **62**: 245–7. https://doi.org/10.1016/j.jadohealth.2017.09.016.
41 Maggs JL, Cassinat JR, Kelly BC, Mustillo SA, Whiteman SD. Parents who first allowed adolescents to drink alcohol in a family context during spring 2020 COVID-19 emergency shutdowns. *J Adolesc Health* 2021; **68**: 816–8. https://doi.org/10.1016/j.jadohealth.2021.01.010.
42 Mares SHW, Van der Vorst H, Engels RCME, Lichtwarck-Aschoff A. Parental alcohol use, alcohol-related problems, and alcohol-specific attitudes, alcohol-specific communication, and adolescent excessive alcohol use and alcohol-related problems: An indirect path model. *Addict Behav* 2011; **36**: 209–16. https://doi.org/10.1016/j.addbeh.2010.10.013.
43 Moser RP, Jacob T. Parent–child interactions and child outcomes as related to gender of alcoholic parent. *J Subst Abuse* 1997; 9: 189–208. https://doi.org/10.1016/S0899-3289(97)90016-X.
44 Ohannessian CM. Parental problem drinking and adolescent psychosocial adjustment: The mediating role of adolescent-parent communication. *J Res Adolesc* 2012; **22**: 498–511. https://doi.org/10.1111/j.1532-7795.2012.00791.x.
45 Rangarajan S. Mediators and moderators of parental alcoholism effects on offspring self-esteem. *Alcohol Alcoholism* 2008; **43**: 481–91. https://doi.org/10.1093/alcalc/agn034.
46 Roberts R, Beckwith M, Watts D. Mothers' intentions to introduce their adolescent to alcohol use: Does mothers' alcohol use effect intentions? *Aust N Z J Public Health* 2010; **34**: 281–7. https://doi.org/10.1111/j.1753-6405.2010.00527.x.
47 Rochat TJ, Houle B, Stein A, Mitchell J, Bland RM. Maternal alcohol use and children's emotional and cognitive outcomes in rural South Africa. *S Afr Med J* 2019; **109**: 526–34. https://doi.org/10.7196/SAMJ.2019.v109i7.13120.
48 Rutherford MJ, Cacciola JS, Alterman AI, McKay JR, Cook TJ. Young men's perceived quality of parenting based on familial history of alcoholism. *J Child Adolesc Subst Abuse* 1998; **6**: 43–56. https://doi.org/10.1300/J029v06n03_03.
49 Schacht PM, Cummings EM, Davies PT. Fathering in family context and child adjustment: A longitudinal analysis. *J Fam Psychol* 2009; **23**: 790–7. https://doi.org/10.1037/a0016741.
50 Senchak M, Leonard KE, Greene BW, Carroll A. Comparisons of adult children of alcoholic, divorced, and control parents in four outcome domains. *Psychol Addict Behav* 1995; **9**: 147–56. https://doi.org/10.1037/0893-164X.9.3.147.
51 Smyth BP, Darker CD, Donnelly-Swift E, Barry JM, Allwright SP. A telephone survey of parental attitudes and behaviours regarding teenage drinking. *BMC Public Health* 2010; **10**: 297. https://doi.org/10.1186/1471-2458-10-297.
52 Spieker SJ, Gillmore MR, Lewis SM, Morrison DM, Lohr MJ. Psychological distress and substance use by adolescent mothers: Associations with parenting attitudes and the quality of mother–child interaction. *J Psychoact Drugs* 2001; **33**: 83–93. https://doi.org/10.1080/02791072.2001.10400472.
53 Su J, Kuo SI, Aliev F, et al. Influence of parental alcohol dependence symptoms and parenting on adolescent risky drinking and conduct problems: A family systems perspective. *Alcohol Clin Exp Res* 2018; **42**: 1783–94. https://doi.org/10.1111/acer.13827.

54	Taber-Thomas SM, Knutson JF. Association between mothers' alcohol use histories and deficient parenting in an economically disadvantaged sample. *Child Maltreat* 2021; **26**: 40–9. https://doi.org/10.1177/1077559520925550.
55	Tweed SH, Ryff CD. Family climate and parent–child relationships: Recollections from a nonclinical sample of adult children of alcoholic fathers. *Res Nurs Health* 1996; **19**: 311–21. https://doi.org/10.1002/(SICI)1098-240X(199608)19:4<311::AID-NUR5>3.0.CO;2-L.
56	Tyrlík M, Konecný S. Moderate alcohol consumption as a mediator of mother's behaviour towards her child. *Cent Eur J Public Health* 2011; **19**: 143–6. https://doi.org/10.21101/cejph.a3665.
57	Van der Vorst H, Engels RC, Meeus W, Deković M. The impact of alcohol-specific rules, parental norms about early drinking and parental alcohol use on adolescents' drinking behavior. *J Child Psychol Psychiatry* 2006; **47**: 1299–306. https://doi.org/10.1111/j.1469-7610.2006.01680.x.
58	Van der Zwaluw CS, Scholte RH, Vermulst AA, Buitelaar JK, Verkes RJ, Engels RC. Parental problem drinking, parenting, and adolescent alcohol use. *J Behav Med* 2008; **31**: 189–200. https://doi.org/10.1007/s10865-007-9146-z.
59	Ward BM, Snow PC. Factors affecting parental supply of alcohol to underage adolescents. *Drug Alcohol Rev* 2011; **30**: 338–43. https://doi.org/10.1111/j.1465-3362.2010.00228.x.
60	Wolf JP, Freisthler B, Chadwick CC. Stress, alcohol use, and punitive parenting during the COVID-19 pandemic. *Child Abuse Negl* 2021; **117**: 105090. https://doi.org/10.1016/j.chiabu.2021.105090.
61	Zhang L, Welte JW, Wieczorek WF. The influence of parental drinking and closeness on adolescent drinking. *J Stud Alcohol* 1999; **60**: 245–51. https://doi.org/10.15288/jsa.1999.60.245.
62	Dumka LE, Roosa MW. The role of stress and family relationships in mediating problem drinking and fathers' personal adjustment. *J Stud Alcohol* 1995; **56**: 528–37. https://doi.org/10.15288/jsa.1995.56.528.
63	Dumka LE, Roosa MW. Factors mediating problem drinking and mothers' personal adjustment. *J Fam Psychol* 1993; **7**: 333–43. https://doi.org/10.1037/0893-3200.7.3.333.
64	Hyphantis T, Koutras V, Liakos A, Marselos M. Alcohol and drug use, family situation and school performance in adolescent children of alcoholics. *Int J Soc Psychiatry* 1991; **37**: 35–42. https://doi.org/10.1177/002076409103700105.
65	Mahato B, Ali A, Jahan M, Verma AN, Singh AR. Parent–child relationship in children of alcoholic and non-alcoholic parents. *Ind Psychiatry J* 2009; **18**: 32–5. https://doi.org/10.4103/0972-6748.57855.
66	Reich W, Earls F, Powell J. A comparison of the home and social environments of children of alcoholic and non-alcoholic parents. *Br J Addict* 1988; **83**: 831–9. https://doi.org/10.1111/j.1360-0443.1988.tb00518.x.
67	Sebre S, Sprugevica I, Novotni A, et al. Cross-cultural comparisons of child-reported emotional and physical abuse: Rates, risk factors and psychosocial symptoms. *Child Abuse Negl* 2004; **28**: 113–27. https://doi.org/10.1016/j.chiabu.2003.06.004.
68	Seilhamer RA, Jacob T, Dunn NJ. The impact of alcohol consumption on parent–child relationships in families of alcoholics. *J Stud Alcohol* 1993; **54**: 189–98. https://doi.org/10.15288/jsa.1993.54.189.
69	Stout ML, Mintz LB. Differences among nonclinical college women with alcoholic mothers, alcoholic fathers, and nonalcoholic parents. *J Couns Psychol* 1996; **43**: 466–72. https://doi.org/10.1037/0022-0167.43.4.466.
70	Zincir H, Yagmur F, Kaya Erten Z, Balci E, Elmali F. The incidence of domestic violence, the causative factors and their effects on the family. *Pak J Med Sci Online* 2010; **2626**: 201–5.
71	Amundsen, V. R., Gjesmoe, A., Andreassen, E., Bølstad, E., Severinsen, Y., Viana, K., Bjørk, R. F., Nygaard, E., Bekkhus, M., & Tsotsi, S. (2025). Alcohol use and parental socialization of emotion in a population-based sample. *Parenting*, *25*(1). https://doi.org/10.1080/15295192.2025.2465988
72	Freisthler, B., Sarabia, J., & Price Wolf, J. (2023). Using ecological momentary assessments to understand how drinking during special occasions relates to parenting behaviors. *Alcohol: Clinical and Experimental Research*, *47*(12), 2343–2353. https://doi.org/10.1111/acer.15206
73	Freisthler, B., & Wolf, J. (2023). Sequencing drinking events and use of punitive, nonpunitive, and positive parenting behaviors with ecological momentary assessment. *Drug and Alcohol Dependence*, *242*, Article 109716. https://doi.org/10.1016/j.drugalcdep.2022.109716
74	Jacques, D. T., Sturge-Apple, M. L., Davies, P. T., & Cicchetti, D. (2025). Maternal alcohol dependence symptoms, maternal insensitivity to children's distress, and young children's blunted emotional reactivity. *Development and Psychopathology*, *37*, 555–577. https://doi.org/10.1017/S0954579424000324
75	Keller, P. S., Rawn, K. P., Dunsmore, J., & Zvolensky, M. (2024). Parental problem drinking, parent emotion socialization and child emotion regulation. *Journal of Applied Developmental Psychology*, *95*, Article 101724. https://doi.org/10.1016/j.appdev.2024.101724

76 Keller, P. S., Rawn, K. P., Dunsmore, J. C., Zvolensky, M., & El-Sheikh, M. (2023). Parental drinking and observations of parent–child problem-solving discussions: Do drinking motives matter? *Journal of Family Psychology*, *37*(7), 993–1004. https://doi.org/10.1037/fam0001135
77 Laslett, A.-M., Kuntsche, S., Wilson, I. M., Taft, A., Fulu, E., Jewkes, R., & Graham, K. (2022). The relationship between fathers' heavy episodic drinking and fathering involvement in five Asia-Pacific countries: An individual participant data meta-analysis. *Alcoholism: Clinical and Experimental Research*, *46*(12), 2137–2148. https://doi.org/10.1111/acer.14955
78 Seay, K. D. (2026). Pathways from parent substance misuse to child and adolescent trauma. *Children and Youth Services Review*, *181*, Article 108724. https://doi.org/10.1016/j.childyouth.2025.108724
79 Wolf, J. P., & Freisthler, B. (2025). Maternal drinking, stress and use of aggressive parenting over the course of the COVID-19 pandemic. *Alcohol and Alcoholism*, *60*(3), agaf020. https://doi.org/10.1093/alcalc/agaf020

Table S4: Studies Excluded on Critical Appraisal

Author (year)	Risk of bias
Dumka & Roosa (1993)[1]	a) c)
Dumka & Roosa (1995)[2]	a) c)
Hyphantis et al. (1991)[3]	e)
Mahato et al. (2009)[4]	a) b) e)
Reich et al. (1988)[5]	a) c) d)
Sebre et al. (2004)[6]	a) b) c)
Seilhamer et al. (1993)[7]	a) c) d)
Stout & Mintz (1996)[8]	a) c) d)
Zincir et al. (2010)[9]	e)

Reasons for the higher risk of bias assessed using the CASP checklists:

a) Selection bias that might compromise the generalizability/validity of the findings: Was the sample recruited in an acceptable way? Was it representative of a defined population? Was there a sufficient number of cases/participants?
b) Measurement or classification bias: Was the exposure/outcome accurately measured to minimize bias?
c) Confounding factors: Have the authors identified all the important confounding factors? Have they taken account of the confounding factors in the design and/or analysis?
d) Are the design and methods of the study appropriate for the research question? Are the results reliable, or could they be due to bias, chance, or confounding?
e) The lack of a description or incomplete/unclear information fundamental for the quality assessment, such as information related to sample recruitment, methods, results, and the measurement of the exposure and outcome variables.

References:

1 Dumka LE, Roosa MW. Factors mediating problem drinking and mothers' personal adjustment. *J Fam Psychol* 1993; **7**: 333–43. https://doi.org/10.1037/0893-3200.7.3.333.

2 Dumka LE, Roosa MW. The role of stress and family relationships in mediating problem drinking and fathers' personal adjustment. *J Stud Alcohol* 1995; **56**: 528–37. https://doi.org/10.15288/jsa.1995.56.528.

3 Hyphantis T, Koutras V, Liakos A, Marselos M. Alcohol and drug use, family situation and school performance in adolescent children of alcoholics. *Int J Soc Psychiatry* 1991; **37**: 35–42. https://doi.org/10.1177/002076409103700105.

4 Mahato B, Ali A, Jahan M, Verma AN, Singh AR. Parent–child relationship in children of alcoholic and non-alcoholic parents. *Ind Psychiatry J* 2009; **18**: 32–5. https://doi.org/10.4103/0972-6748.57855.

5 Reich W, Earls F, Powell J. A comparison of the home and social environments of children of alcoholic and non-alcoholic parents. *Br J Addict* 1988; **83**: 831–9. https://doi.org/10.1111/j.1360-0443.1988.tb00518.x.

6 Sebre S, Sprugevica I, Novotni A, et al. Cross-cultural comparisons of child-reported emotional and physical abuse: Rates, risk factors and psychosocial symptoms. *Child Abuse Negl* 2004; **28**: 113–27. https://doi.org/10.1016/j.chiabu.2003.06.004.

7 Seilhamer RA, Jacob T, Dunn NJ. The impact of alcohol consumption on parent–child relationships in families of alcoholics. *J Stud Alcohol* 1993; **54**: 189–98. https://doi.org/10.15288/jsa.1993.54.189.

8 Stout ML, Mintz LB. Differences among nonclinical college women with alcoholic mothers, alcoholic fathers, and nonalcoholic parents. *J Couns Psychol* 1996; **43**: 466–72. https://doi.org/10.1037/0022-0167.43.4.466.

9 Zincir H, Yagmur F, Kaya Erten Z, Balci E, Elmali F. The incidence of domestic violence, the causative factors and their effects on the family. *Pak J Med Sci Online* 2010; **2626**: 201–5.
